# The association between antiretroviral therapy and selected cardiovascular disease risk factors in sub-Saharan Africa: A systematic review and meta-analysis

**DOI:** 10.1371/journal.pone.0201404

**Published:** 2018-07-30

**Authors:** Christian Akem Dimala, Hannah Blencowe, Simeon Pierre Choukem

**Affiliations:** 1 Department of Infectious Disease Epidemiology, London School of Hygiene and Tropical Medicine, London, United Kingdom; 2 Department of Orthopaedics, Southend University Hospital, Essex, United Kingdom; 3 Health and Human Development (2HD) Research Network, Douala, Cameroon; 4 Diabetes and Endocrinology Unit, Department of Internal Medicine, Douala General Hospital, Douala, Cameroon; 5 Department of Internal Medicine and Paediatrics, Faculty of health Sciences, University of Buea, Buea, Cameroon; University of Perugia, ITALY

## Abstract

**Background:**

With increasing adverse cardiovascular disease (CVD) outcomes in HIV/AIDS patients, the possible contribution of antiretroviral therapy (ART) to the prevailing CVD epidemic in sub-Saharan Africa (SSA) through its effect on CVD risk factors has rather been under investigated. This study aimed to assess the extent to which ART is associated with hypertension, diabetes mellitus (DM) and dyslipidemia in SSA.

**Methods:**

This is a systematic review and meta-analysis of studies from SSA, published between January 1946 and December 2017, from Medline, Embase, Africa-wide Information, the Cochrane library, African Index and Medicus databases. Both observational and interventional studies with comparable ART-treated and ART-naïve populations were selected and data was extracted from eligible studies. Pooled estimates of the effect of ART on the outcomes of interest (hypertension, diabetes and abnormal lipid profiles) were obtained using random effects meta-analysis, and meta-regression analysis was used to explore between-study heterogeneity.

**Results:**

Twenty cross-sectional studies were included involving 5386 participants. There was no association between ART use and hypertension (OR: 1.9, 95%CI: 0.96–3.76, n = 8, I^2^ = 73.8%) and DM (OR: 2.53, 95%CI: 0.87–7.35, n = 8, I^2^ = 73.8%). ART use was associated with high total cholesterol (OR: 3.85, 95%CI: 2.45–6.07, n = 8, I^2^ = 67.0%), high triglycerides (OR: 1.46, 95%CI: 1.21–1.75, n = 14, I^2^ = 10.0%) and high LDL-cholesterol (OR: 2.38, 95%CI: 1.43–3.95, n = 11, I^2^ = 87.6%). ART was associated with rather lower odds of having low HDL-cholesterol (OR: 0.53, 95%CI: 0.32–0.87, n = 8, I^2^ = 78.2%). There was evidence of between-study heterogeneity for all outcomes except high triglycerides.

**Conclusions:**

ART appears to be associated with CVD risk in HIV/AIDS patients in SSA only through dyslipidemia but not through hypertension and DM, however, high quality and robust research in SSA is mandated to accurately ascertain the actual contribution of ART to the CVD burden in this part of the world. Nevertheless, HIV/AIDS patients should still benefit from systematic CVD screening alongside their regular care services.

**Trial registration:**

**Prospero Registration** - CRD42016042306.

## Introduction

Sub-Saharan Africa (SSA) remains the region most affected by HIV/AIDS with an estimated 25.5 million cases, 1.4 million new infections and 0.8 million AIDS-related deaths in 2015 [1]. The introduction of antiretroviral therapy (ART) and the continuous scale up of its coverage to about 46% globally and 47% in SSA in 2015, has significantly helped to reduce AIDS-related morbidity and mortality over the years, with a 45% global reduction in deaths since 2005 [1]. Cardiovascular disease (CVD), which collectively refers to the diseases involving the heart and blood vessels, is a major public health problem, accounting for 37% of all non-communicable disease-related deaths globally in 2012 [2]. Contrary to HIV/AIDS, the CVD epidemic has been steadily on the rise in Low and Middle-Income countries (LMICs) with over three quarters of global CVD-related deaths occurring in these countries [2]. This CVD epidemic is largely due to the increasing incidence of its major risk factors; hypertension (HTN); diabetes mellitus (DM), and abnormal blood lipid levels (high total cholesterol (TC), high serum triglycerides (TG), low high density lipoprotein cholesterol (HDL) and high low density lipoprotein cholesterol (LDL)) [3]. Of more concern is the increasingly observed higher CVD occurrences in the HIV-infected population compared to HIV negative control subjects [4–6], and the higher CVD rates in ART-treated compared to ART-naïve patients, suggestive of a probable association between ART and these CVD risk factors [4–13]. However, assessing the association between ART and CVD risk factors has been markedly confounded by the prevailing epidemiological transition towards rising incidence and prevalence of non-communicable diseases. A systematic review on this probable association between ART and CVD among HIV-infected patients in SSA found significantly higher rates of adverse cardio-metabolic traits in patients on ART compared to ART-naïve individuals, attributing this difference to ART use [5]. However, this review compared the standardized mean differences in blood pressure, blood glucose and lipid profile parameters between the various patient groups and was not actually on hypertension, diabetes or dyslipidemia respectively, which are more precise and relevant measures of adverse disease outcomes. Moreover, more studies especially in SSA with varying conclusions on this subject have been published after the former review. This study was therefore conducted to systematically review and summarize published studies on the extent to which exposure to ART is associated to these CVD risk factors in SSA, given the specificities of HIV/AIDS and access to ART in the SSA region. We had as specific objectives to estimate overall measures of effect for the association between ART and hypertension, diabetes mellitus and dyslipidemia (elevated total cholesterol, triglycerides and low density lipoprotein cholesterol and reduced high density lipoprotein cholesterol) respectively in HIV/AIDS patients in SSA.

## Methods

### Search strategy and selection criteria

This was a systematic review and meta-analysis of published studies on the association between ART use and the selected CVD risk factors (hypertension, diabetes mellitus, and dyslipidemia).

The search was completed in December 2017 and the following databases were searched: Medline (1946–2017), Embase (1947–2017) Africa-wide Information (1960–2017), the Cochrane library (1996–2017) and African Index Medicus (2002–2017). The search was centered around the 3 key concepts; anti-retroviral therapy; cardiovascular disease risk factors (hypertension, diabetes, dyslipidemia) and sub-Saharan Africa (each sub-Sahara African country included). For each key concept, the subject heading search was combined to the free-text search of the synonyms and derivatives of the main concept using the boolean operator ‘OR’, then these results from the 3 key words were combined all together using the boolean operator ‘AND’. The search strategy conducted for Ovid Medline and Ovid Embase is presented in S1 Table. Articles returned by the search were saved on the Zotero version 4.0.29.10 reference manager software for removal of duplicates and screening of titles and abstracts of articles. The full text of the remaining articles were then reviewed for eligibility in accordance with the selection criteria by two investigators (CAD and HB).

The following studies were included:

- Cross-sectional, cohort, case-control and randomized controlled trials with data on the prevalence or incidence of hypertension, DM or lipid profile disorders in HIV/AIDS patients.
- Studies with comparable ART-treated and ART-naïve populations.
- Studies published in English up to July 2017 in the selected databases.
- Studies involving participants aged 18 and above, living in one of the countries in SSA.

Studies excluded were:

- Unpublished manuscripts and conference abstracts.
- Studies that only compared the mean blood pressure, glucose and lipid levels in the ART-treated and ART-naïve groups rather than the actual prevalence or incidence of hypertension, DM and dyslipidemia.
- Studies from which we could not calculate the relevant CVD risk factor parameters or the measures of effect from the provided data
- Studies with diagnostic criteria and cut-off values for hypertension, DM and abnormal lipid profiles different from those internationally recognized.
- Same studies published in different journals with the same or a different title.

For cohort studies with several publications of their results over time, the most recent of the studies was used. The reference lists of relevant articles were also searched for eligible studies.

### Data analysis

Data were abstracted from eligible studies onto the pre-tested data abstraction form produced on Epi info version 7.1.0.6 statistical software (CDC, Atlanta, USA) by the principal investigator (CAD) and reviewed by a second investigator (HB). The following data were abstracted: First author, publication year, journal, study period, study design and setting, socio-demographic and clinical information of the participants such as mean or median age, sex distribution, duration of HIV infection and of ART use, ART regimens, the measures of disease frequency and effect for the cardiovascular risk factors (hypertension, DM and abnormal lipid profiles) in the ART and ART-naïve groups. When not directly available, the appropriate measures of effect such as the odds ratios for the outcomes were derived from the reported data and entered into the abstraction form. The data were doubled-checked for errors and accuracy after completion of abstraction and exported to STATA version 14.1 statistical software for analysis.

The exposure variable was ART use, categorized into: ART and ART-naïve groups for patients who had ever received ART and those who had never received ART respectively. The outcome variables were the presence of hypertension, diabetes and abnormal lipid profiles/dyslipidemia (high TC, high TG, low HDL-cholesterol and high LDL-cholesterol) respectively. These outcomes were defined according to their respective standard International criteria (S2 Table). The odds ratio was used as the measure of effect to compare the outcome in the ART and ART-naïve groups. Crude odds ratios were derived from the raw data extracted from the studies and adjusted odds ratios were preferentially used when reported. Pooled estimates of the odds ratios for the outcomes comparing the ART and ART-naïve groups were derived using random effects meta-analysis and presented on Forest plots showing both the size and direction of the overall effect. The Cochran’s Q test was performed for evidence of heterogeneity across studies with low P values suggestive of between-study heterogeneity. The I^2^ test statistic was also used to assess the degree of heterogeneity among the studies, reported as the percentage of between-study variability not due to chance [14]. The Harbord’s test and the Peter’s test [15], were used to statistically assess for funnel plot asymmetry and small-study effects. Meta-regressions and subgroup stratified analyses were also done according to the study quality, study location, sample size and adjusting of confounders or not, to explore potential sources of heterogeneity among the studies.

All studies were assessed for their quality and risk of bias using the Quality Assessment Tool for Observational Cohort and Cross-Sectional Studies of the National Health Institute (National Heart, Lung, and Blood Institute) and given an overall rating of good, fair or poor based on well-defined criteria [16]. Studies were assessed for their research questions and objectives, their study populations, sample sizes and participation rates, the validity, reliability and consistency in the measurement of exposure and outcome variables among study participants, and the measurement and adjustment of potential confounders (S3 Table).

The Grading of Recommendations Assessment, Development and Evaluation (GRADE) approach was also used to assess the quality of evidence with respect to each specific outcome [17]. The overall quality of evidence on these various outcomes was graded as high, moderate, low or very low (S4 Table).

This review was reported in accordance with the Preferred Reporting Items for Systematic Reviews and Meta-Analysis (PRISMA) 2009 guidelines (S5 Table). Prospero Registration—CRD42016042306

The Research Governance and Integrity Office of the Research Ethics Committee of the London School of Hygiene and Tropical Medicine reviewed the study protocol and assessed the study as not requiring an ethical approval (LSHTM Ethics Reference: 11026).

## Results

**Fig 1:**
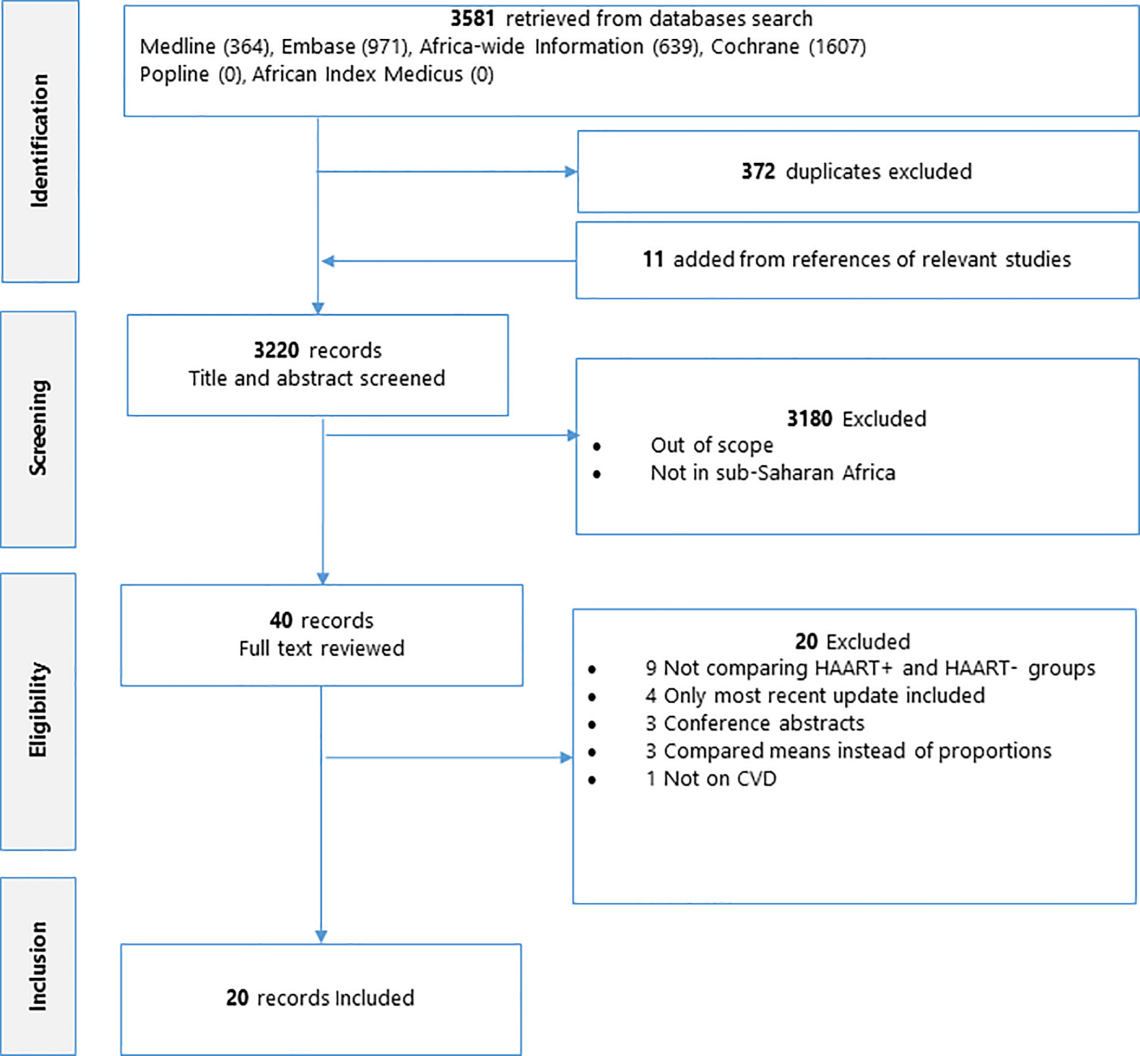
PRISMA flow chart detailing the study selection process.

Fig 1 presents a flow chart of the study selection process, with the number of articles retrieved at each stage from the search results through the title and full-text review to the studies finally included in the review. The search strategy returned a total of 3581 articles, to which 11 studies were added from the search of the reference list of relevant studies, and 372 duplicates were removed. From the 3220 records left, 3180 were excluded since they were out of scope and not in sub-Saharan Africa, leaving 40 potentially eligible studies for full text review. Twenty studies were subsequently excluded based on the selectin criteria (12 not comparing ART and ART-naïve groups, 4 with more recent updates of their findings, 3 conference abstracts, 3 comparing means rather than proportions of the outcome measures, and 1 not on CVD risk factors). Twenty studies were finally included in the analysis [13,18–36]. These studies included a total of 5386 participants (Table 1). A general summary of all the included studies and the 20 excluded studies including the reasons for their exclusion are presented on S6 and S7 Tables respectively. All the studies included were cross-sectional in design and participants were recruited in hospital settings. There was no difference in the overall mean percentage of males (30.7±8.1 vs 34.4±9, p = 0.271, n = 14), mean BMI (23.9±1.3 vs 23.1±1.5, p = 0.124, n = 16) and CD4 cell count (382±59 vs 322±100, p = 0.073, n = 13) between the ART and the ART-naïve groups respectively. The group on ART was older than the ART-naïve group (mean age: 39.2±3.5 vs 36.8±3.4 years, p = 0.042, n = 18). The median of the mean or median duration of ART use in the respective studies was 38 months (interquartile range: 30–56). Overall 9 studies were rated as being of good quality and 11 as being of fair quality based on the assessment criteria (S3 Table). The GRADE evidence profile for the assessed outcomes has been presented on S4 Table. Overall, the quality of evidence on high TC, low HDL-cholesterol and high LDL-cholesterol was moderate given the overall strengths of the associations observed, while that on hypertension, DM and high TG were rated as low given the combinations of inconsistencies, publication bias (high TG) and weak overall strengths of association. Graphical evidence of publication bias for the respective outcomes have been presented on S1–S6 Figs.

**Table 1:** Socio-demographic characteristics of the participants from the eligible studies.

#	Author (Year)	Country	Study Years	Parti-cipants	Males (%)	Age in years*	ART duration (months)*
1	Abebe (2014)	Ethiopia	2011–2012	252	27.8	35.3	-
2	Awotedu (2010)	South Africa	2009–2010	196	18.9	36.9	-
3	Ayodele (2012)	Nigeria	-	291	32.6	39.5	17.2
4	Botha (2014)	South Africa	-	137	-	43.0	34.2
5	Dave (2011)	South Africa	-	849	-	-	16.0
6	Dimala (2016)	Cameroon	2013	200	30.0	39.1	58.6
7	Ekali (2013)	Cameroon	-	143	28.0	39.5	-
8	Maganga (2015)	Tanzania	-	301	-	-	56.0
9	Manuthu (2008)	Kenya	2006	295	42.0	-	-
10	Mbunkah (2014)	Cameroon	2010–2011	173	28.9	38.7	-
11	Mohammed (2015)	Ethiopia	2014	393	33.1	-	-
12	Muhammad (2013)	Nigeria	2009	200	47.0	32.5	-
13	Ngala (2013)	Ghana	2009–2010	305	-	-	-
14	Nsagha (2015)	Cameroon	2014	215	25.1	43.2	62.7
15	Ogundahunsi (2008)	Nigeria	-	110	-	-	-
16	Ogunmola (2014)	Nigeria	-	250	37.6	37.6	34.1
17	Osegbe (2016)	Nigeria	-	200	-	36.6	-
18	Pefura Yone (2011)	Cameroon	2009–2010	276	39.1	39.6	30.2
19	Tadewos (2012)	Ethiopia	2012	226	35.0	35.5	49.4
20	Tesfaye (2014)	Ethiopia	2012–2013	374	33.7	-	42.6

* Mean/median

**Fig 2:**
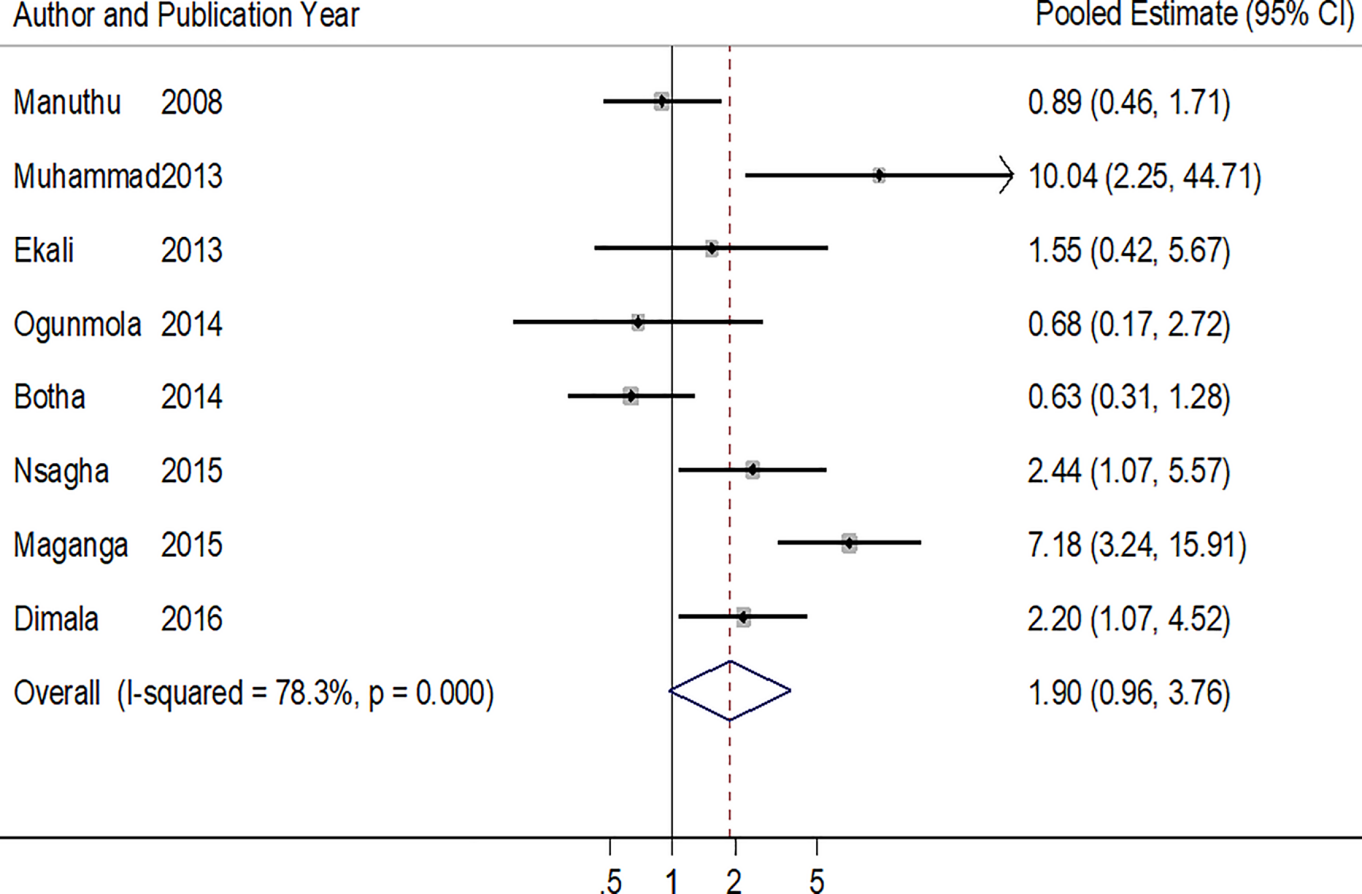
Forest plot of the studies reporting the association between ART use and hypertension. The solid line on the Forest plot is the point of no effect (OR = 1) and the dashed line represents the overall pooled estimate. The grey squares and horizontal lines represent the odds ratios of each study and their 95% confidence intervals. The size of the grey square represents the weight contributed by each study in the meta-analysis. The diamond represents the pooled odds ratio and its 95% confidence intervals.

Eight studies reported on the association between ART use and hypertension [13,21,23–25,28,30,32]. Of these studies, only 4 had strong evidence of a significant association, all in favor of higher odds of HTN in patients on ART [13,24,28,30]. The pooled estimate suggests the absence of any statistically significant association between ART use and HTN (OR: 1.90, 95% CI: 0.96–3.76, p = 0.065) (Table 2 & Fig 2). The studies were heterogeneous (p<0.001) with a 73.8% variation across studies (I^2^). There was weak evidence of publication bias statistically (Harbord’s test p = 0.926 and Peter’s test p = 0.403).

**Table 2:** Individual and pooled odds ratios of the studies reporting the association between ART use and hypertension.

Author (Year)	Proportion of ART+ with HTN (%)	Proportion of ART-naïve with HTN (%)	Crude /Adjusted Odds Ratio (95% CI)	P value
Manuthu (2008)	18/134 (13.4)	24/161 (14.9)	0.89 (0.46–1.71)	0.425
Muhammad (2013)	17/100 (17.0)	2/100 (2.0)	10.03 (2.25–44.71)	**<0.001**
Ekali (2013)	18/115 (15.7)	3/28 (10.7)	1.55 (0.42–5.67)	-
Ogunmola (2014)	16/130 (12.3)	19/120 (15.8)	0.68* (0.17–2.72)	0.587
Botha (2014)	20/66 (30.3)	29/71 (40.8)	0.63 (0.31–1.28)	0.280
Nsagha (2015)	47/160 (29.4)	8/55 (14.5)	2.44 (1.07–5.57)	**0.033**
Maganga (2015)	43/150 (28.7)	8/151 (5.3)	7.183 (3.24–15.91)	-
Dimala (2016)	38/100 (38.0)	19/100 (19.0)	2.20* (1.07–4.52)	**0.032**
**Pooled Estimate Heterogeneity P<0.001, I^2^ = 78.3%**			**1.90 (0.96–3.76)**	0.065
**Heterogeneity P<0.001, I^2^ = 78.3%**				

* Adjusted odd ratios obtained from the logistic regression models of the respective studies.

**Fig 3:**
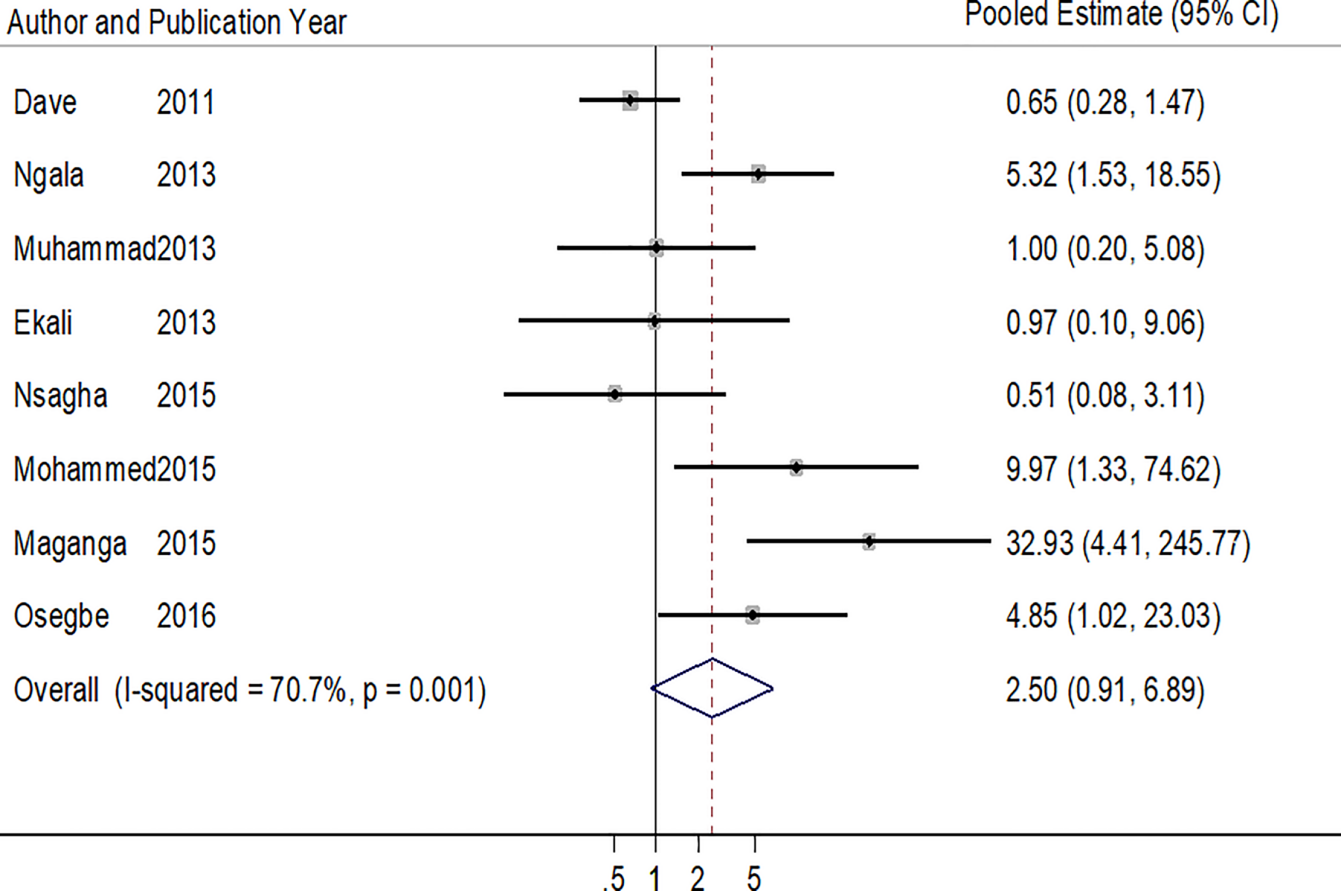
Forest plot of the studies reporting the association between ART use and diabetes mellitus. The solid line on the Forest plot is the point of no effect (OR = 1) and the dashed line represents the overall pooled estimate. The grey squares and horizontal lines represent the odds ratios of each study and their 95% confidence intervals. The size of the grey square represents the weight contributed by each study in the meta-analysis. The diamond represents the pooled odds ratio and its 95% confidence intervals.

Eight studies reported on DM [22–24,27–30,33], and 3 of these studies found statistically higher odds of DM in patients on ART (Table 3 & Fig 3) [27,29,33]. Overall, there was no statistically significant association between DM and ART use (Pooled estimate: 2.53, 95% CI: 0.87–7.35, p = 0.089), and a significant between-study heterogeneity (p<0.001, I^2^ statistic = 70.7%). There was no statistical evidence of publication bias (Harbord’s test p = 0.347 and Peter’s test p = 0.788).

**Table 3:** Pooled and individual odds ratios of the studies reporting the association between ART use and diabetes mellitus.

Author (year)	Proportion of ART+ with DM (%)	Proportion of ART- with DM (%)	Crude/Adjusted Odds Ratio (95% CI)	P value
Dave (2011)	10/443 (2.3)	14/406 (3.4)	0.66 (0.28–1.47)	0.292
Ngala (2013)	17/164 (10.4)	3/141 (2.1)	5.32 (1.53–18.56)	**0.007**
Muhammad (2013)	3/100 (3.0)	3/100 (3.0)	1.00 (0.20–5.08)	1.000
Ekali (2013)	4/115 (3.4)	1/28 (3.6)	0.973 (0.10–9.06)	0.610
Nsagha (2015)	3/160 (1.9)	2/55 (3.6)	0.51 (0.08–3.11)	0.463
Mohammed (2015)	24/284 (8.5)	1/109 (0.9)	9.97 (1.33–74.62)	**0.006**
Maganga (2015)	27/150 (18.0)	1/151 (0.7)	32.93 (4.41–245.77)	-
Osegbe (2016)	9/100 (9.0)	2/100 (2.0)	4.86 (1.02–23.03)	**0.030**
**Pooled Estimate**			**2.53 (0.87–7.35)**	0.089
**Heterogeneity P<0.001, I^2^ = 70.7%**				

**Fig 4:**
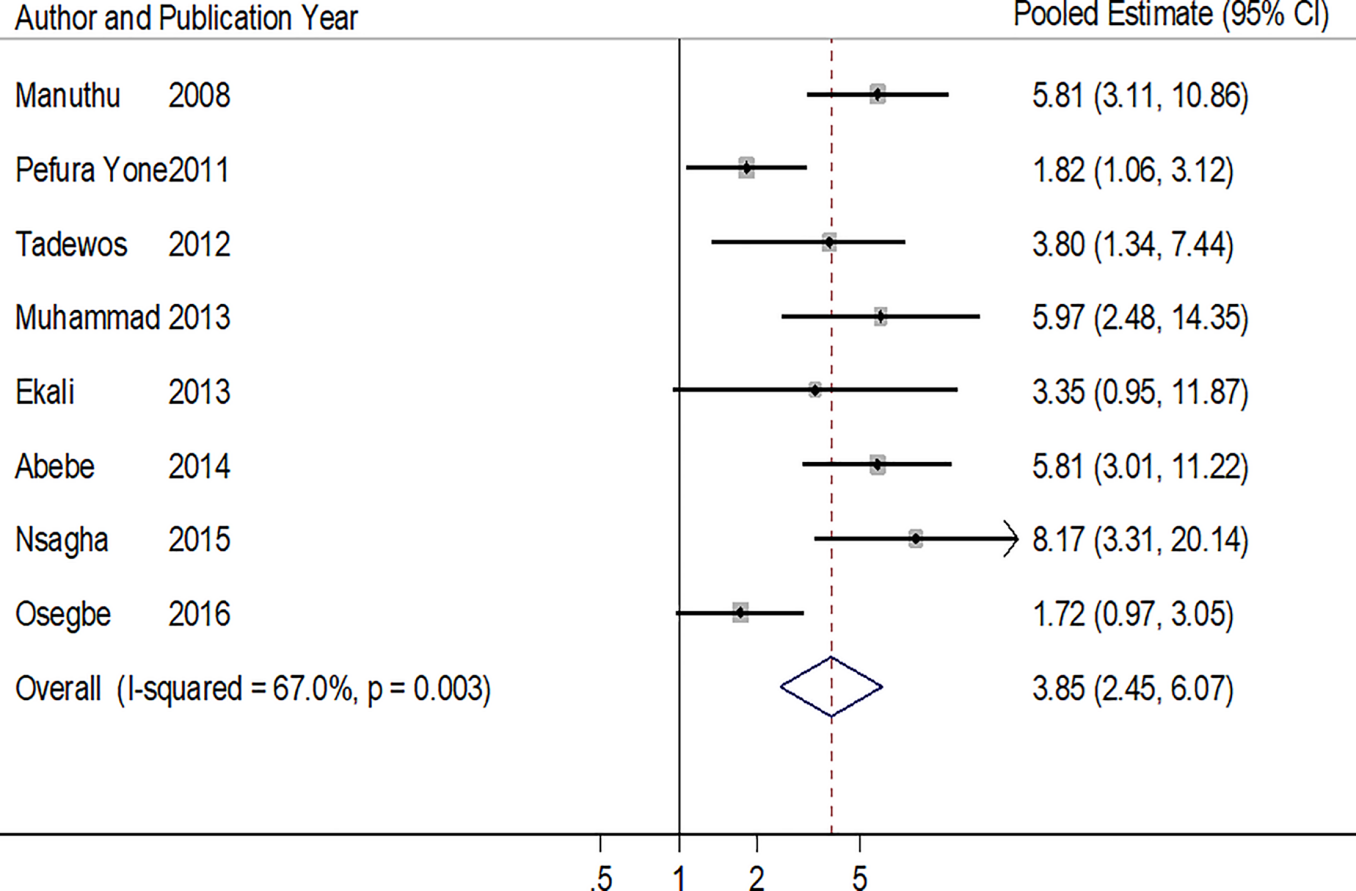
Forest plot of the studies reporting the association between ART use and high total cholesterol. The solid line on the Forest plot is the point of no effect (OR = 1) and the dashed line represents the overall pooled estimate. The grey squares and horizontal lines represent the odds ratios of each study and their 95% confidence intervals. The size of the grey square represents the weight contributed by each study in the meta-analysis. The diamond represents the pooled odds ratio and its 95% confidence intervals.

All 8 studies that reported on total cholesterol found higher odds of having a high TC level in patients receiving ART compared to ART-naïve patients [18,23,25,28,30,33–35]. The overall pooled estimate was 3.85 (95% CI: 2.45–6.07, p<0.001), the between study heterogeneity p = 0.003 and I^2^ statistic = 67.0%. (Table 4 & Fig 4) and the Harbord’s test and the Peter’s test p values for small study effect were 0.740 and 0.871 respectively.

**Table 4:** Pooled and individual odds ratios of the studies reporting the association between ART use and high total cholesterol.

Author (year)	Proportion of ART+ with high TC (%)	Proportion of ART- with high TC (%)	Crude/Adjusted Odd ratios (95% CI)	P value
Manuthu (2008)	51/134 (38.1)	16/161 (9.9)	5.81 (3.10–10.9)	**<0.001**
Pefura Yone (2011)	52/138 (37.7)	33/138 (23.9)	1.82* (1.06–3.12)	**0.020**
Tadewos (2012)	49/113 (43.4)	18/113 (15.9)	3.8* (1.34–7.44)	**<0.001**
Muhammad (2013)	31/100 (31.0)	7/100 (7.0)	5.97 (2.48–14.35)	**<0.001**
Ekali (2013)	33/115 (28.7)	3/28 (10.7)	3.35 (0.95–11.87)	-
Abebe (2014)	53/126 (42.0)	14/126 (11.1)	5.82 (3.00–11.22)	**<0.001**
Nsagha (2015)	80/160 (50.0)	6/55 (10.9)	8.17 (3.31–20.14)	**<0.001**
Osegbe (2016)	47/100 (47.0)	34/100 (34.0)	1.72 (0.97–3.05)	0.060
**Pooled Estimate**			**3.85 (2.45–6.07)**	**<0.001**
**Heterogeneity P = 0.003, I^2^ = 67.0%**				

* Adjusted odd ratios obtained from the logistic regression models of the respective studies.

**Fig 5:**
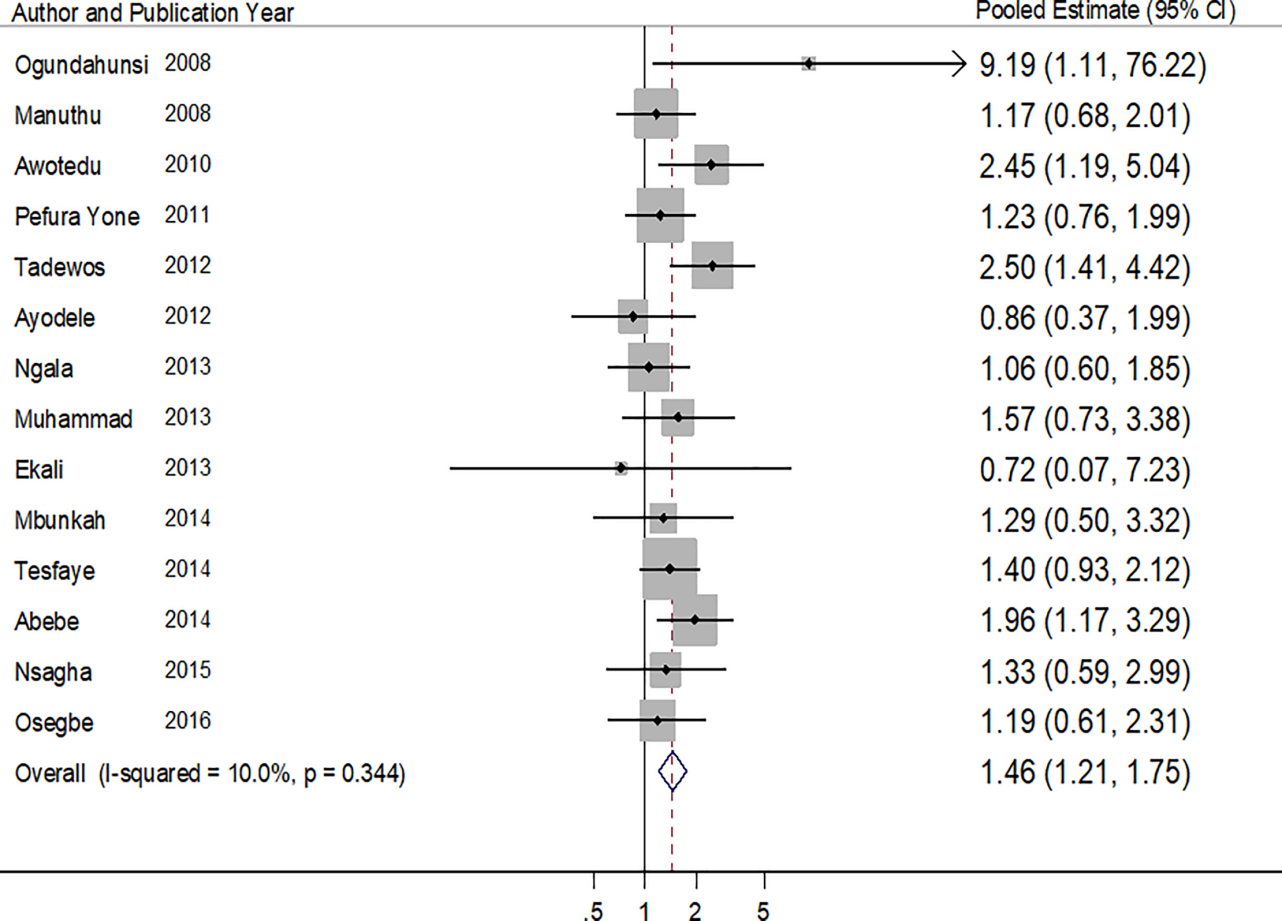
Forest plot of the studies reporting the association between ART use and high serum triglyceride. The solid line on the Forest plot is the point of no effect (OR = 1) and the dashed line represents the overall pooled estimate. The grey squares and horizontal lines represent the odds ratios of each study and their 95% confidence intervals. The size of the grey square represents the weight contributed by each study in the meta-analysis. The diamond represents the pooled odds ratio and its 95% confidence intervals.

Fourteen studies reported on the association between ART use and high TG [18–20,23,25,26,28–31,33–36]. The pooled estimate was in favor of an association between ART use and high TG (OR: 1.46, 95% CI: 1.21–1.75, p<0.001) (Table 5 & Fig 5). There was no evidence of heterogeneity among the studies (p = 0.344, I^2^ statistic = 10.0%).There was some evidence of publication bias (Harbord’s and Peter’s test p values of 0.909 and 0.052 respectively).

**Table 5:** Pooled and individual odds ratios of the studies reporting the association between ART use and high serum triglyceride.

Author (year)	Proportion of ART+ with high TG (%)	Proportion of ART- with high TG (%)	Crude/Adjusted Odd ratios (95% CI)	P value
Ogundahunsi (2008)	8/55 (14.5)	1/55 (1.8)	9.19 (1.11–76.23)	-
Manuthu (2008)	33/134 (24.6)	36/161 (22.3)	1.18 (0.69–2.04)	-
Awotedu (2010)	24/86 (27.9)	15/110 (13.6)	2.45 (1.19–5.03)	-
Pefura Yone (2011)	60/138 (43.5)	53/138 (38.4)	1.23 (0.76–2.00)	0.392
Tadewos (2012)	63/113 (55.8)	35/113 (31.0)	2.5* (1.41–4.42)	**0.002**
Ayodele (2012)	8/236 (3.4)	8/55 (14.5)	0.86 (0.37–1.99)	0.716
Ngala (2013)	34/164 (20.7)	28/141 (19.9)	1.1 (0.60–1.85)	0.829
Muhammad (2013)	19/100 (19.0)	13/100 (13.0)	1.57 (0.73–3.38)	0.247
Ekali (2013)	3/115 (2.6)	1/28 (3.6)	0.43 (0.07–0.72)	0.630
Mbunkah (2014)	16/112 (14.3)	7/61 (11.5)	1.29 (0.50–3.32)	-
Tesfaye (2014)	85/188 (45.2)	69/186 (37.1)	1.40 (0.93–2.11)	-
Abebe (2014)	59/126 (46.8)	39/126 (31.0)	1.96 (1.19–3.29)	**0.010**
Nsagha (2015)	33/160 (20.6)	9/55 (16.4)	1.33 (0.59–2.99)	0.493
Osegbe (2016)	24/100 (24.0)	21/100 (21.0)	1.19 (0.61–2.31)	0.610
**Pooled Estimate**			**1.46 (1.21–1.75)**	**<0.001**
**Heterogeneity P = 0.344, I^2^ = 10.0%**				

* Adjusted odd ratios obtained from the logistic regression models of the respective studies.

**Fig 6:**
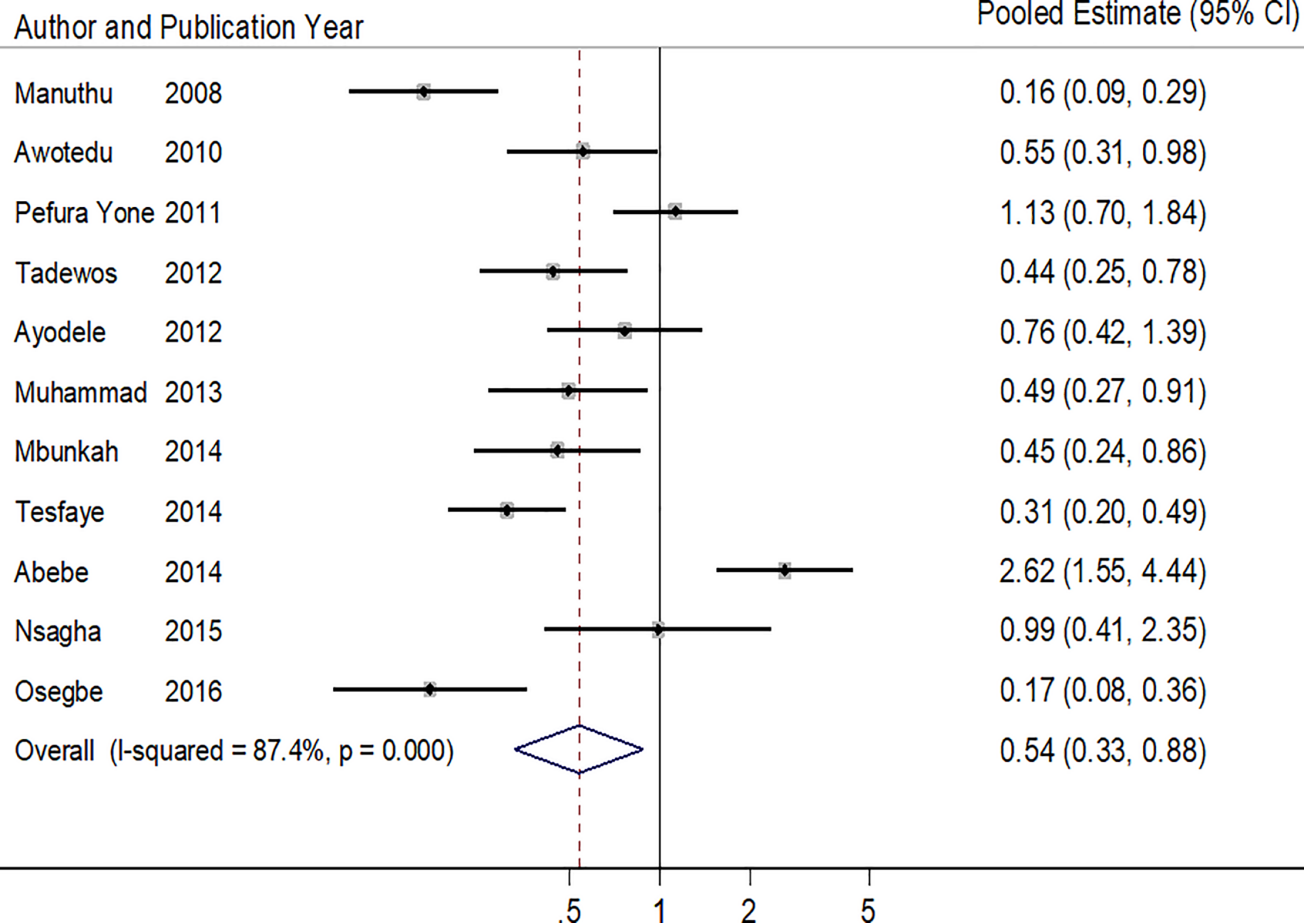
Forest plot of the studies reporting the association between ART use and low HDL-cholesterol. The solid line on the Forest plot is the point of no effect (OR = 1) and the dashed line represents the overall pooled estimate. The grey squares and horizontal lines represent the odds ratios of each study and their 95% confidence intervals. The size of the grey square represents the weight contributed by each study in the meta-analysis. The diamond represents the pooled odds ratio and its 95% confidence intervals.

Of the 11 studies that reported on low HDL-cholesterol [18–20,25,26,28,30,33–36], all but two of them found higher odds of low HDL-cholesterol in ART-naïve patients compared to patients on ART [18,34], The pooled estimate was 0.53 (95% CI: 0.3 2–0.87, p = 0.014) and there was between-study heterogeneity (p<0.001, I^2^ statistic = 87.4%) (Table 6 & Fig 6) but no statistical evidence of publication bias (Harbord’s test P = 0.765 and Peter’s test = 0.936).

**Table 6:** Pooled and individual odds ratios of the studies reporting the association between ART use and low HDL-cholesterol.

Author (year)	Proportion of ART+ with low HDLc (%)	Proportion of ART- with low HDLc (%)	Crude/Adjusted Odd ratios (95% CI)	P value
Manuthu (2008)	19/134 (0.14)	82/161 (50.9)	0.16 (0.09–0.29)	**<0.001**
Awotedu (2010)	44/86 (51.2)	72/110 (65.5)	0.55 (0.31–0.99)	-
Pefura Yone (2011)	55/138 (39.9)	51/138 (37.0)	1.13 (0.70–1.84)	0.620
Tadewos (2012)	49/113 (0.43)	72/113 (63.7)	0.44* (0.25–0.78)	**0.005**
Ayodele (2012)	126/236 (0.53)	33/55 (60.0)	0.76 (0.42–1.39)	0.786
Muhammad (2013)	61/100 (61.0)	76/100 (76.0)	0.49 (0.27–0.91)	**0.022**
Mbunkah (2014)	50/112 (44.6)	39/61 (63.9)	0.46 (0.24–0.86)	-
Tesfaye (2014)	101/188 (53.7)	147/186 (79.0)	0.31 (0.20–0.49)	-
Abebe (2014)	62/126 (49.2)	34/126 (27.0)	2.62 (1.55–4.44)	**0.001**
Nsagha (2015)	23/160 (14.4)	8/55 (14.5)	0.99 (0.41–2.35)	0.975
Osegbe (2016)	11/100 (11.0)	42/100 (42.0)	0.17 (0.08–0.36)	**0.001**
**Pooled Estimate**			**0.53 (0.32–0.87)**	**0.014**
**Heterogeneity P<0.001, I^2^ = 87.4%**				

* Adjusted odd ratios obtained from the logistic regression models of the respective studies.

**Fig 7:**
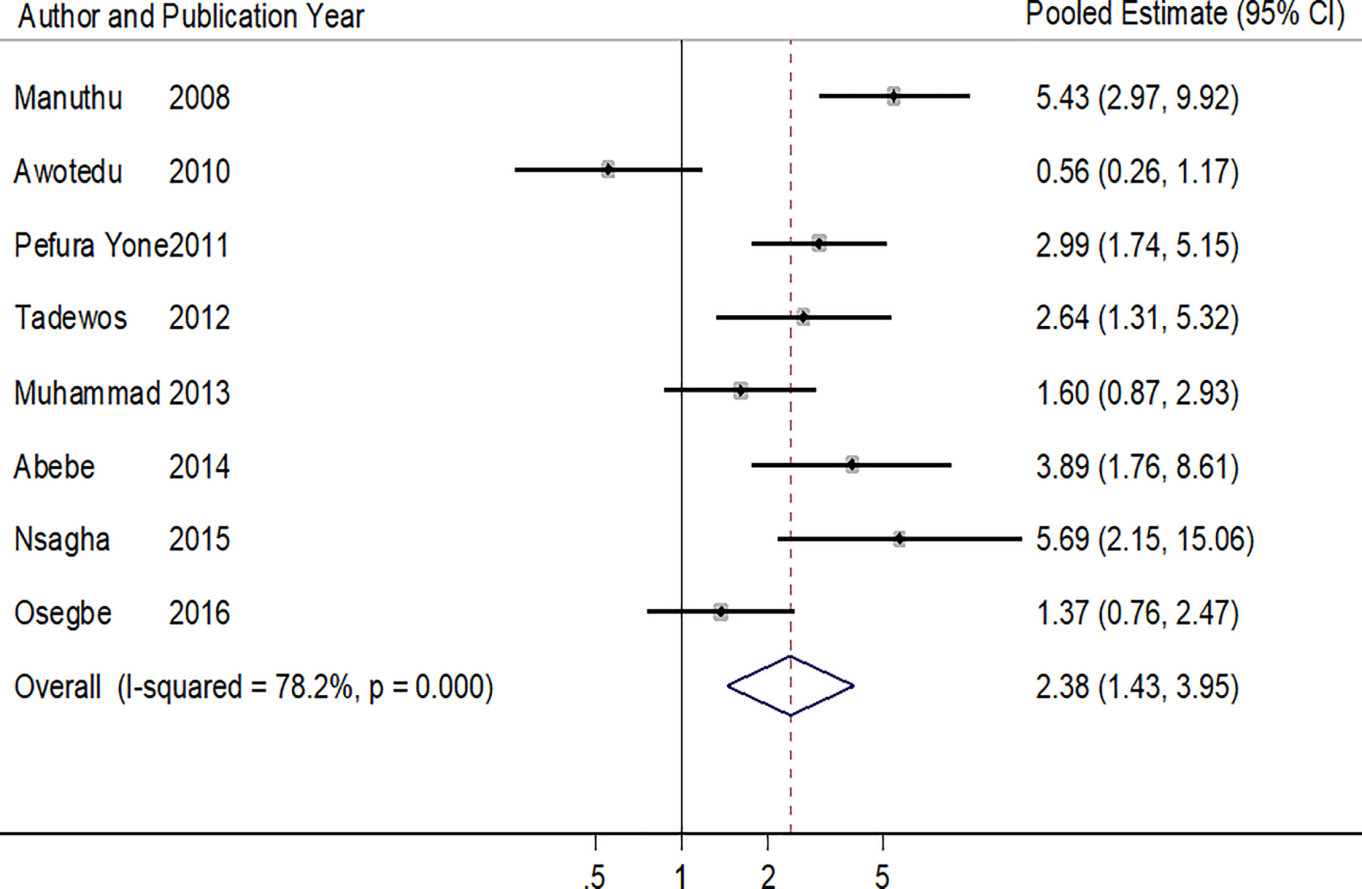
Forest plot of the studies reporting the association between ART use and high LDL-cholesterol. The solid line on the Forest plot is the point of no effect (OR = 1) and the dashed line represents the overall pooled estimate. The grey squares and horizontal lines represent the odds ratios of each study and their 95% confidence intervals. The size of the grey square represents the weight contributed by each study in the meta-analysis. The diamond represents the pooled odds ratio and its 95% confidence intervals.

The pooled estimate from the 8 studies with reports on high LDL-cholesterol [18,19,25,28,30,33–35], was suggestive of an association between high LDL-cholesterol and ART use (OR:2.38, 95%CI: 1.43–3.95, p = 0.001) (Table 7 & Fig 7). There was strong evidence of study heterogeneity (p = 0.001), with a 78.2% (I^2^) total variation across studies. The Harbord’s and Peter’s test P values were 0.909 and 0.052 respectively.

**Table 7:** Pooled and individual odds ratios of the studies reporting the association between ART use and high LDL-cholesterol.

Author (year)	Proportion of ART+ with high LDLc (%)	Proportion of ART- with high LDLc (%)	Crude/Adjusted Odd ratios (95% CI)	P value
Manuthu (2008)	53/134 (39.6)	18/161 (11.1)	5.43 (2.97–9.92)	**<0.001**
Awotedu (2010)	67/86 (77.9)	95/110 (86.4)	0.56 (0.26–1.17)	-
Pefura Yone (2011)	64/138 (46.4)	29/138 (21.0)	2.99* (1.74–5.15)	**<0.001**
Tadewos (2012)	38/113 (33.6)	17/113 (15.0)	2.64* (1.31–5.32)	**0.006**
Muhammad (2013)	36/100 (36.0)	26/100 (26.0)	1.60 (0.87–2.93)	0.126
Abebe (2014)	29/126 (23.0)	9/126 (7.1)	3.89 (1.76–8.61)	**<0.001**
Nsagha (2015)	58/160 (36.3)	5/55 (9.1)	5.69 (2.15–15.06)	**<0.001**
Osegbe (2016)	37/100 (37.0)	30/100 (30.0)	1.37 (0.76–2.47)	0.290
**Pooled Estimate**			**2.38 (1.43–3.95)**	**0.001**
**Heterogeneity P<0.001, I^2^ = 78.2%**				

* Adjusted odd ratios obtained from the logistic regression models of the respective studies.

Study quality was categorized as good and fair, study location categorized into West or Central Africa and East or Southern Africa, sample size grouped as ≥ 250 and < 250 participants, and lastly studies were categorized as having adjusted for confounders or not. The between-stratum and within-stratum heterogeneity P values and I^2^ statistics were derived. Comparing the ART to the ART-naïve group, the pooled estimate was higher among studies of good quality compared to those of fair quality for all measured outcomes except for DM (meta-regression relative OR:0.38), suggesting a stronger association between ART use and the outcomes in studies of good quality. On the other hand the pooled estimate was lower in studies that adjusted for confounders compared to those that did not for, except for high TG and high LDL-cholesterol (meta-regression relative OR:1.84 and 1.12 respectively). Overall, there was no statistical evidence that the between-study heterogeneity could be explained by any of these study characteristics (S8 Table).

## Discussion

This study aimed to assess the extent to which exposure to ART is associated with selected CVD risk factors in SSA by reviewing published studies with measures of effect on this association and deriving corresponding pooled estimates of these measures through meta-analysis. Contrary to the previous studies that reported on an association between ART and hypertension and/or diabetes, this review found no significant direct association between hypertension, diabetes and ART, and suggests this observation could rather be due to the dyslipidemia associated with ART use.

Though patients on ART were found to have a 90% higher odd of having hypertension compared to their ART naïve counterparts, the association between ART and hypertension from the aggregated 8 studies was not statistically significant. It is worth noting that one of the four studies that found no association between ART and hypertension, however, reported that the prevalence of hypertension significantly increased as treatment duration increased [23]. As such, the inclusion of patients on ART for very short durations in that study and the inclusion of such studies with low median ART durations in our analyses could have contributed to diminishing the observed effect of ART on blood pressure. This was earlier mentioned by studies which suggested that the effects of ART on blood pressure are best appreciated after prolonged exposure to ART [37,38]. There was no evidence of publication bias and the meta-regression analysis exploring the observed heterogeneity between the studies did not find any particular study characteristic that could explain this inconsistency in studies. However, the sub-group analysis was limited to study quality, study location, sample size and adjustment for confounders and did not include important parameters such as age and duration of ART use since they were not provided by all the studies. The review by Dillon *et al* conducted in SSA had findings similar to those of this review with no association between ART use and higher blood pressure values [5]. This finding is, however, contrary to that of a more recent review by Nduka *et al* which found a significantly higher risk of hypertension associated with ART use worldwide [12]. Just a quarter of the studies in the latter review were from SSA with contrasting findings on this association. The effect of ART on blood pressure should be considered and addressed together with other factors such as environmental factors, genetic and lifestyle predispositions to hypertension.

There was also no significant association between ART use and DM. This finding is similar to that earlier reported by Dillon *et al* who did not observe any association between ART use and fasting blood glucose levels in SSA [5]. Some large prospective studies have reported an association between ART and DM [10,39,40]. and the studies by Brown *et al*[10] and Tien *et al* [39] had HIV-uninfected patients as control subjects making it challenging to attribute the observed association solely to ART use given the fact that HIV infection itself has been associated to some extent to DM [41]. Also the large cohort study by De Wit *et al* [40] consisted of participants from Europe, South and North America and Australia but not SSA raising up the question if the effect of ART on glucose metabolism varies with ethnicity. Even though the studies with evidence of no association between ART use and DM were of higher quality than those that found an association, the meta-regression analysis did not identify the studies’ quality as a possible explanation for the heterogeneity in the studies.

There was a strong association between ART use and abnormal lipid parameters implying that patients on treatment are at a greater risk of having these CVD risk factors. These findings are largely consistent with those of a recent review associating ART to dyslipidemia [42]. Dillon *et al* in the review in SSA found no association between ART and high TG, and explained this was probably due to the relatively greater use of NNRTI-based regimes compared to PI-based regimens, known to induce more dyslipidemia [42]. The studies reporting on these lipid disorders with the exception of high TG, were heterogeneous and the meta-regression did not identify any study characteristic that could explain this observation. This actual heterogeneity among studies could therefore be a possible explanation for the funnel plots asymmetry, since statistical evidence for small-study effects and publication bias in these cases were inconclusive.

Most studies did not report the exact proportions of patients on specific ART regimens to enable us to assess associations between individual ART agents and the respective CVD risk factors but many more studies reported the use of NNRTIs over PIs as is currently the case with first-line regimens in SSA. Associations of greater magnitude could therefore be observed in settings with predominantly PI-based regimens which have been identified as inducing more extreme dyslipidemic profiles compared to NNRTIs [42–44].

Several mechanisms have been proposed as possible pathways through which ART could lead to this multitude of metabolic abnormalities including chronic inflammation due to the HIV infection itself, increased circulating inflammatory markers and cytokines involved in insulin and lipid regulation [45,46], but several unanswered questions still remain with regards to the exact contribution of ART to CVD. The association of ART to particular CVD risk factors instead of others invariably suggests that the overall effect of ART in HIV/AIDS patients depends on the complex interplay and combination of several factors such as genetic and environmental predispositions.

It is worth noting that all the studies of this review are limited by their cross-sectional design. It is not possible to detect if all patients developed hypertension, DM or abnormal lipid profiles before or after initiating ART making it challenging to ascertain any causality between ART use and these outcomes. This therefore makes the quality of evidence on these outcomes low to moderate at best, and ensuing recommendations of low strength. However, the study aimed to identify any possible association irrespective of its nature, and reverse causality in this case is less likely since the presence of hypertension, DM or dyslipidemia is unlikely to result in ART use. Selectively exposing patients to ART and monitoring for outcomes that could take years to occur is even more challenging and involves enormous ethical and logistic considerations. Such intervention studies are therefore difficult to come by, leaving cross-sectional studies as the primary sources of evidence to answer these research questions.

Some studies had small sizes with no statistical justifications for these, implying they had greater sampling errors and lower statistical power to detect actual associations that might have occurred not merely due to chance. This makes their findings less reliable. In addition to this, not all studies reported the durations of ART among their participants, and there were differences in ART duration among studies that reported these, increasing the between-study heterogeneity. Also few studies adjusted for the major confounders either at the methodological or the statistical phases, making residual confounding a possible explanation for the observed associations. Despite these limitations, this study remains important as one of the few systematic reviews and meta-analysis providing epidemiological, clinical and public health data on HIV/AIDS and CVD in SSA. An extensive search strategy was used and several databases searched making the review exhaustive on the subject.

In conclusion, contrary to the previous reports of an association between hypertension, diabetes and ART, this review suggests there is no direct association between hypertension and diabetes. The associations often observed between ART and these CVD risk factors could be as a result of its association to dyslipidemia (hypercholesterolemia, hypertriglyceridemia, high LDL-cholesterol blood levels) as found in this review. This review, however, exposes the utmost need of high quality and robust research on this subject in SSA to ascertain the actual impact of ART on CVD risk factors and the contribution of specific ART agents to adverse CVD outcomes. Nevertheless, HIV/AIDS patients should still benefit from systematic CVD screening for CVD risk factors alongside their regular counselling and care services.

## Supporting information

S1 Table Search strategy for Ovid Medline and Ovid Embase databases. (PDF)

S2 Table International criteria for defining outcome measures. (PDF)

S3 Table Quality assessment tool for observational cohort and cross-sectional studies for the 20 eligible studies. (PDF)

S4 Table GRADE evidence profile for the measured outcomes. (PDF)

S5 Table Preferred Reporting Items for Systematic Reviews and Meta-Analysis (PRISMA) 2009 Checklist. (PDF)

S6 Table General overview and characteristics of the included studies. (PDF)

S7 Table Studies excluded from the review and reasons for exclusion. (PDF)

S8 Table Sub-group analyses and meta-regression for the respective outcomes. (PDF)

S1 Fig Funnel Plot for studies on the association between HAART use and hypertension with log odds ratios displayed on the horizontal scale. The blue dots represent the studies, the solid vertical line represents the log odds ratio of the pooled estimate obtained from the meta-analysis, the dashed diagonal lines represent the 95% confidence limits around the pooled estimate.
(TIF)

S2 Fig Funnel plot for studies on the association between HAART use and diabetes mellitus with log odds ratios displayed on the horizontal scale. The blue dots represent the studies, the solid vertical line represents the log odds ratio of the pooled estimate obtained from the meta-analysis, the dashed diagonal lines represent the 95% confidence limits around the pooled estimate.
(TIF)

S3 Fig Funnel plot for studies on the association between HAART use and high total cholesterol with log odds ratios on the horizontal scale. The blue dots represent the studies, the solid vertical line represents the log odds ratio of the pooled estimate obtained from the meta-analysis, the dashed diagonal lines represent the 95% confidence limits around the pooled estimate.
(TIF)

S4 Fig Funnel plot for studies on the association between HAART use and high triglycerides respectively with log odds ratios on the horizontal scale. The blue dots represent the studies, the solid vertical line represents the log odds ratio of the pooled estimate obtained from the meta-analysis, the dashed diagonal lines represent the 95% confidence limits around the pooled estimate.
(TIF)

S5 Fig Funnel plot for studies on the association between HAART use and low HDL-cholesterol with log odds ratios displayed on the horizontal scale. The blue dots represent the studies, the solid vertical line represents the log odds ratio of the pooled estimate obtained from the meta-analysis, the dashed diagonal lines represent the 95% confidence limits around the pooled estimate.
(TIF)

S6 Fig Funnel plot for studies on the association between HAART use and high LDL-cholesterol respectively with log odds ratios displayed on the horizontal scale. The blue dots represent the studies, the solid vertical line represents the log odds ratio of the pooled estimate obtained using the fixed effects meta-analysis, the dashed diagonal lines represent the 95% confidence limit around the pooled estimate.
(TIF)

## Abbreviations

ADA: American Diabetes Association
AIDS: Acquired Immuno-Deficiency Syndrome
ART: Antiretroviral Therapy
BMI: Body Mass Index
CI: Confidence Interval
CD4: Cluster of Differentiation 4
CDC: Centre for Disease Control and Prevention
DM: Diabetes Mellitus
GRADE: Grading of Recommendations Assessment, Development and Evaluation
HDL: High Density Lipoprotein
HIV: Human Immuno-Deficiency Syndrome
HTN: Hypertension
IDF: International Diabetes Federation
JNC: Joint National Committee
LDL: Low Density Lipoprotein
LIMC: Low and Middle Income Country
OR: Odds Ratio
NCEP/ATP: National Cholesterol Education Program/Adult Treatment Panel
NNRTI: Non-Nucleoside Reverse Transcriptase Inhibitors
NRTI: Nucleoside Reverse Transcriptase Inhibitors
PI: Protease Inhibitor
PRISMA: Preferred Reporting Items for Systematic Reviews and Meta-Analysis
SSA: Sub-Saharan Africa
TC: Total Cholesterol
TG: Triglycerides
WHO: World Health organization
