# Supplementary material for: The association between antiretroviral therapy and selected cardiovascular disease risk factors in sub-Saharan Africa: A systematic review and meta-analysis
Source: PLoS One. 2018 Jul 30;13(7):e0201404. doi: 10.1371/journal.pone.0201404 (PMC6066235; doi:10.1371/journal.pone.0201404)
Supplement: S1 Table — (PDF) [file pone.0201404.s001.pdf]

S1 Table. Search strategy for Ovid Medline and Ovid Embase databases

Search #	Search words
**1**	Antiretroviral therapy, Highly Active [MeSH terms]
**2**	Highly active antiretroviral therapy OR antiretroviral therapy OR antiretroviral OR HAART OR ART OR anti-retroviral OR Nucleotide reverse transcriptase inhibitor OR NRTI OR Non-nucleoside reverse transcriptase inhibitor OR NNRTI OR Protease inhibitor OR PI OR PIs OR lopinavir OR ritonavir OR lamivudine OR zidovudine OR stavudine OR nevirapine OR efavirenz OR tenofovir OR emtricitabine OR atazanavir OR darunavir
**3**	#1 OR #2
**4**	Cardiovascular diseases [MeSH terms]
**5**	Hypertension OR high blood pressure OR systolic blood pressure OR diastolic blood pressure OR SBP OR DBP OR diabetes mellitus OR diabetes OR type 2 diabetes OR type 2 diabetes mellitus OR diabetic OR type 2 diabetic OR dysglycemia OR dysglycaemia OR hyperglycemia OR hyperglycaemia OR glucose OR insulin resistance OR insulin OR hyperinsulinemia OR hyperinsulinaemia OR dyslipidemias OR dyslipidemia OR hyperlipidemia OR hypercholesterolemia OR hypertriglyceridemia OR cholesterol OR triglyceride OR triglycerides OR HDL OR LDL OR VLDL OR hyperlipoproteinemia OR lipoprotein OR hyperlipidaemia OR hypercholesterolaemia OR hypertriglyceridaemia
**6**	#4 OR #5
**7**	Sub-Saharan Africa [MeSH terms]
**8**	Africa OR Algeria OR Angola OR Benin OR Botswana OR Burkina Faso OR Burundi OR Cameroon OR Canary Islands OR Cape Verde OR Central African Republic OR Chad OR Comoros OR Congo OR Democratic Republic of Congo OR Djibouti OR Egypt OR Equatorial Guinea OR Eritrea OR Ethiopia OR Gabon OR Gambia OR Ghana OR Guinea OR Guinea Bissau OR Ivory Coast OR Cote dIvoire OR Jamahiriya OR Jamahiryia OR Kenya OR Lesotho OR Liberia OR Libya OR Libia OR Madagascar OR Malawi OR Mali OR Mauritania OR Mauritius OR Mayotte OR Morocco OR Mozambique OR Mocambique OR Namibia OR Niger OR Nigeria OR Principe OR Reunion OR Rwanda OR Sao Tome OR Senegal OR Seychelles OR Sierra Leone OR Somalia OR South Africa OR St Helena OR Sudan OR Swaziland OR Tanzania OR Togo OR Tunisia OR Uganda OR Western Sahara OR Zaire OR Zambia OR Zimbabwe OR Central Africa OR Central African OR West Africa OR West African OR Western Africa OR Western African OR East Africa OR East African OR Eastern Africa OR Eastern African OR North Africa OR North African OR Northern Africa OR Northern African OR South African OR Southern Africa OR Southern African OR subSaharan Africa OR subSaharan African OR sub-Saharan Africa OR sub-Saharan African
**9**	#7 OR #8
**10**	#3 AND #6 AND #9

MeSH – Medical Subject Heading
