# Supplementary material for: The association between antiretroviral therapy and selected cardiovascular disease risk factors in sub-Saharan Africa: A systematic review and meta-analysis
Source: PLoS One. 2018 Jul 30;13(7):e0201404. doi: 10.1371/journal.pone.0201404 (PMC6066235; doi:10.1371/journal.pone.0201404)
Supplement: S2 Table — (PDF) [file pone.0201404.s002.pdf]

S2 Table. International Criteria for Defining Outcome Measures

Outcome	Criteria	Organization	Author (Publication Year)
**Hypertension**	SBP ≥ 140 and/or<br>DBP ≥ 90 mmHg	WHO/JNC 7	Botha (2014), Dimala (2016),<br>Ekali (2013), Maganga (2015),<br>Manuthu (2008), Muhammad (2013)<br>Nsagha (2015), Ogunmola (2014)
**Diabetes**	FBG ≥ 126 mg/dL<br>(7.0 mmol/L)<br>OR<br>RBG ≥ 200 mg/dL<br>(11.1 mmol/L)	WHO/ADA	Dave (2011), Ekali (2013),<br>Maganga (2015), Mohammed (2015),<br>Muhammad (2013), Ngala (2013),<br>Nsagha (2015), Osegbe (2016)
**High TC**	Serum TC ≥ 200 mg/dL<br>(5.17 mmol/L)	NCEP/ATP III	Abebe (2014), Ekali (2013)<br>Manuthu (2008), Muhammad (2013)<br>Nsagha (2015), Pefura Yone (2011)<br>Tadewos (2012), Osegbe (2016)
**High TG**	Serum TG ≥ 200 mg/dL<br>(2.23 mmol/L)	NCEP/ATP II	Ekali (2013)<br>Ogunhadunsi (2008)
	Serum TG ≥ 150 mg/dL<br>(1.70 mmol/L)	NCEP/ATP III	Abebe (2014), Awotedu (2010),<br>Ayodele (2012), Manuthu (2008)<br>Mbunkah (2014), Muhammad (2013)<br>Nsagha (2015), Pefura Yone (2011)<br>Tesfaye (2014), Tadewos (2012)<br>Osegbe (2016), Ngala (2013)
**Low HDLc**	Men:<br>Serum HDL < 40 mg/dL<br>(1.03 mmol/L)<br>Women:<br>Serum HDL < 50 mg/dL<br>(1.29 mmol/L)	NCEP/ATP III	Abebe (2014), Awotedu (2010)<br>Ayodele (2012), Manuthu (2008)<br>Mbunkah (2012), Muhammad (2013)<br>Nsagha (2015), Pefura Yone (2011)<br>Tesfaye (2014), Tadewos (2012)<br>Osegbe (2016)
**High LDLc**	Serum LDL ≥ 130 mg/dL<br>(3.36 mmol/L)	NCEP/ATP III	Abebe (2014), Pefura Yone (2011)<br>Osegbe (2016), Manuthu (2008)
	Serum LDL ≥ 100 mg/dL<br>(2.59 mmol/L)	NCEP/ATP III	Awotedu (2010), Muhammad (2013)<br>Nsagha (2015), Tadewos (2012)

SBP – Systolic blood pressure, DBP – Diastolic blood pressure, WHO – World Health Organization
JNC 7 - 7th report of the Joint National Committee on the prevention, detection, evaluation and treatment of high blood pressure, ADA – American Diabetes Association.
NCEP/ATP III - National Cholesterol Education Program/Adult Treatment Panel guidelines III
NCEP/ATP II - National Cholesterol Education Program/Adult Treatment Panel guidelines II
TG – Triglycerides, TC – Total Cholesterol, HDLc – High Density Lipoprotein Cholesterol, LDLc – Low Density Lipoprotein Cholesterol
