# Supplementary material for: The association between antiretroviral therapy and selected cardiovascular disease risk factors in sub-Saharan Africa: A systematic review and meta-analysis
Source: PLoS One. 2018 Jul 30;13(7):e0201404. doi: 10.1371/journal.pone.0201404 (PMC6066235; doi:10.1371/journal.pone.0201404)
Supplement: S3 Table — (PDF) [file pone.0201404.s003.pdf]

S3 Table. Quality Assessment Tool for Observational Cohort and Cross-Sectional Studies for the 20 eligible studies

Criteria	Abebe (2014)	Awotedu (2010)	Ayodele (2012)	Botha (2014)	Dave (2011)	Dimala (2016)	Ekali (2013)	Maganga (2015)	Manuthu (2008)	Mbunkah (2014)
1. Clear research questions/objectives	Yes	Yes	Yes	Yes	Yes	Yes	Yes	No	Yes	No
2. Clearly defined study population	Yes	Yes	Yes	Yes	Yes	Yes	Yes	Yes	Yes	Yes
3. Participation rate ≥ 50%	Yes	Yes	Yes	Yes	Yes	Yes	Yes	Yes	Yes	Yes
4. Selection from same population	Yes	Yes	Yes	Yes	Yes	Yes	Yes	Yes	Yes	Yes
5. Justified sample size and study power	No	No	No	No	No	Yes	No	No	No	No
6. Exposures measured prior to outcomes	Yes	Yes	No	Yes	Yes	Yes	Yes	Yes	Yes	Yes
7. Reasonable timeframe for effect	Yes	No	NR	NR	Yes	Yes	No	Yes	No	No
8. Different levels of exposure	No	No	No	No	No	No	Yes	No	No	No
9. Clearly defined exposure measures	Yes	Yes	Yes	Yes	Yes	Yes	Yes	Yes	Yes	Yes
10. Repeated measure of exposure	No	No	No	No	No	No	No	No	No	No
11. Clearly defined outcomes	Yes	Yes	Yes	Yes	Yes	Yes	Yes	Yes	Yes	Yes
12. Blinded outcome assessors	NR	NR	NR	NR	NR	NR	NR	NR	NR	NR
13. Loss to follow-up ≤ 20%	NA	NA	NA	NA	No	NA	NA	NA	NA	NA
14. Measured and adjusted confounders	No	No	No	No	No	Yes	Yes	Yes	No	No
**Overall Rating**	**Fair**	**Fair**	**Fair**	**Fair**	**Fair**	**Good**	**Good**	**Fair**	**Fair**	**Fair**

**CD - cannot determine, NA - not applicable, NR - not reported*

Continued…

S3 Table Continued…

**Criteria**	**Moha-mmed (2015)**	**Muha-mmad (2013)**	**Ngala (2013)**	**Nsagha (2015)**	**Ogunda-hunsi (2008)**	**Ogun-mola (2014)**	**Osegbe (2016)**	**Pefura Yone (2011)**	**Tadewos (2012)**	**Tesfaye (2014)**
1. Clear research questions/objectives	Yes	Yes	Yes	Yes	No	Yes	Yes	Yes	Yes	Yes
2. Clearly defined study population	Yes	Yes	Yes	Yes	Yes	Yes	Yes	Yes	Yes	Yes
3. Participation rate ≥ 50%	Yes	Yes	Yes	Yes	Yes	Yes	Yes	Yes	Yes	Yes
4. Selection from same population	Yes	Yes	Yes	Yes	Yes	Yes	Yes	Yes	Yes	Yes
5. Justified sample size and study power	Yes	No	No	No	No	No	No	Yes	No	Yes
6. Exposures measured prior to outcomes	Yes	Yes	Yes	Yes	No	Yes	Yes	Yes	Yes	Yes
7. Reasonable timeframe for effect	NR	Yes	Yes	Yes	Yes	No	NR	NR	Yes	Yes
8. Different levels of exposure	Yes	No	Yes	Yes	No	No	No	No	No	Yes
9. Clearly defined exposure measures	Yes	Yes	Yes	Yes	Yes	Yes	Yes	Yes	Yes	Yes
10. Repeated measure of exposure	No	No	No	No	Yes	No	No	No	No	No
11. Clearly defined outcomes	Yes	Yes	Yes	Yes	Yes	Yes	Yes	Yes	Yes	Yes
12. Blinded outcome assessors	NR	NR	NR	NR	NR	NR	NR	NR	NR	NR
13. Loss to follow-up ≤ 20%	NA	NA	NA	NA	No	NA	NA	NA	NA	NA
14. Measured and adjusted confounders	Yes	Yes	No	Yes	No	Yes	No	Yes	Yes	Yes
**Overall Rating**	**Good**	**Good**	**Fair**	**Good**	**Fair**	**Good**	**Fair**	**Good**	**Good**	**Good**

**CD - cannot determine, NA - not applicable, NR - not reported*
