# Supplementary material for: The association between antiretroviral therapy and selected cardiovascular disease risk factors in sub-Saharan Africa: A systematic review and meta-analysis
Source: PLoS One. 2018 Jul 30;13(7):e0201404. doi: 10.1371/journal.pone.0201404 (PMC6066235; doi:10.1371/journal.pone.0201404)
Supplement: S4 Table — (PDF) [file pone.0201404.s004.pdf]

S4 Table. GRADE evidence profile for the measured outcomes

**Quality assessment of evidence on measured outcomes**						**Summary of findings**			
						**Outcome/ Total Participants**			
**Studies (design) (Participants)**	**Limitation[1]**	**Inconsistency[2]**	**Indirectness**	**Imprecision**	**Publication bias[3]**	**HAART**	**HAART naive**	**Pooled Odds ratio**	**Overall Quality**
**Hypertension**									
8 (cross-sectional) (1736)	Serious limitations	Serious Inconsistency	No indirectness	No Imprecision	Undetected	217/951	112/785	1.90 (0.96 - 3.76)	⊕⊕OO Low
**Diabetes Mellitus**									
8 (cross-sectional) (2606)	Serious limitations	Serious Inconsistency	No indirectness	No imprecision	Undetected	97/1516	27/1090	2.53 (0.87 - 7.35)	⊕⊕OO Low
**High total cholesterol**									
8 (cross-sectional) (1802)	Serious limitations	Serious Inconsistency	No indirectness	No imprecision	Undetected	396/982	131/820	3.85 (2.45 - 6.07)	⊕⊕⊕O Moderate
**High triglycerides**									
14 (cross-sectional) (3251)	Serious limitations	No serious inconsistency	No indirectness	No imprecision	Detected	491/1823	335/1428	1.46 (1.21 – 1.75)	⊕⊕OO Low
**Low HDL**									
11 (cross-sectional) (2693)	Serious limitations	Serious Inconsistency	No indirectness	No imprecision	Undetected	596/1489	656/1204	0.53 (0.32 - 0.87)	⊕⊕⊕O Moderate
**High LDL**									
8 (cross-sectional) (1855)	Serious limitations	Serious Inconsistency	No indirectness	No imprecision	Detected	382/953	229/902	2.38 (1.43 - 3.95)	⊕⊕⊕O Moderate

[1]No adjustments for confounders and no justification of sample sizes.

[2]Significant heterogeneity and variability (I^2) except for total cholesterol.

[3]Staticical and graphical evidence of publication bias for high triglycerides and LDL cholesterol.
