# Supplementary material for: The association between antiretroviral therapy and selected cardiovascular disease risk factors in sub-Saharan Africa: A systematic review and meta-analysis
Source: PLoS One. 2018 Jul 30;13(7):e0201404. doi: 10.1371/journal.pone.0201404 (PMC6066235; doi:10.1371/journal.pone.0201404)
Supplement: S6 Table — (PDF) [file pone.0201404.s006.pdf]

S6 Table. General Overview and Characteristics of the included studies

Author (Year) Country	Study aim(s)	Participants, settings, sampling and methods	Outcomes/findings	Limitations	Conclusions
Abebe (2014) Ethiopia	To assess antiretroviral treatment associated hyperglycemia and dyslipidemia among HIV infected patients	Enrollment of 126 patients on HAART and 126 HAART-naïve attending the Burayu Health Center to compare the prevalence of the outcomes	Total cholesterol, Triglyceride<br>High density lipoprotein<br>Low density lipoprotein<br>Blood glucose	Confounders measured but not adjusted.	First-line HAART was associated with atherogenic lipid profile levels which should be monitored regularly in HIV infected patients
Awotedu (2010) South Africa	To determine the prevalence of metabolic syndrome (MS) and insulin resistance (IR) and their determinants in HAART, HAART-naïve and HIV negative patients.	110 on HAART, 86 HAART-naïve and 125 HIV negative controls in Eastern Province. Prevalence of MS and IR compared between the groups.	Metabolic syndrome (central obesity, elevated blood pressure, elevated blood glucose, low HDL and high triglyceride)<br>Insulin resistance	No Dual Energy Xray Absorptiometry (DEXA) scanning to assess body fat proportions and insulin sensitivity.	Prevalence of MS was similar across groups. HIV patients on HAART and MS were independently associated with IR
Ayodele (2012) Nigeria	To determine the prevalence and clinical correlates of metabolic syndrome (MS) in HIV patients. To determine if HAART use and CD4 count level were associated with metabolic syndrome	226 on HAART compared to 55 HAART-naive patients recruited at the Ladoke Akintola University of Technology Teaching Hospital (LTH)	Metabolic syndrome (central obesity, elevated blood pressure, elevated blood glucose, low HDL and high triglyceride)	Small number of male participants.<br>Non-generalizability of finding from urban to rural setting<br>6% with incomplete data.	The prevalence of metabolic syndrome varied with the diagnostic criteria used and MS correlates with traditional cardiovascular risk factors rather than HAART-related factors
Botha (2014) South Africa	To determine whether receiving antiretroviral treatment changes the cardio-metabolic profile of HIV-infected South Africans	The cardio-metabolic profile in a 66 HAART patients was compared to that of 71 HAART-naïve patients in the North-West province of South Africa	Blood pressure (hypertension, change in pulse pressure)<br>Lipid profile parameters	Small sample size.	Compared to HAART-naïve those on HAART had a greater increase in pulse pressure and systolic blood pressure, and unfavorable lipid profile

Continued…

S6 Table continued…

Author (Year) Country	Study aim(s)	Participants, settings, sampling and methods	Outcomes/findings	Limitations	Conclusions
Dave (2011) South Africa	To examine the prevalence and associated risk factors of dysglycemia and insulin sensitivity in HIV-infected South Africans.	Parameters of 446 patients on nonnucleoside reverse transcriptase inhibitor–based HAART and 406 HAART-naïve patients were compared	Dysglycemia<br>Body mass index, skin fold thickness<br>b-cell function	Short duration of exposure to HAART. Few number of males<br>Exclusion of patients with diabetes already.	Both HAART and HAART-naïve patients had a similar prevalence of dysglycemia. Efavirenz was found to be associated with dysglycemia
Dimala (2016) Cameroon	To compare the prevalence of HTN in HAART and HAART-naïve patients and to assess other socio-demographic and clinical factors associated with hypertension	100 patients on HAART were compared to 100 HAART-naïve patients both recruited at the Limbe Regional Hospital HIV treatment center. Blood pressure and other factors were assessed	Hypertension	Not designed to determine HAART agent associated with hypertension.<br>Potential confounders such as diabetes not measured.	The prevalence of hypertension was found to be higher in patients on HAART than in HAART-naïve patients, necessitating regular blood pressure and cardiovascular risk factors monitoring
Ekali (2013) Cameroon	To assess the relationship between HAART duration and cardiometabolic disorders in HIV-infected Cameroonians.	115 patients on HAART were compared to 28 HAART-naïve patients at an HIV treatment center. Blood glucose, blood pressure and anthropometric parameters were assessed	Diabetes<br>Hypertension<br>Insulin sensitivity<br>Abnormal lipid levels	Compliance to treatment not assessed.<br>Small sample size.	HAART duration is associated with obesity, fat distribution, blood pressure and cholesterol levels in HIV-infected Cameroonians,<br>but does not affect glucose metabolism significantly
Maganga (2015) Tanzania	To compare the prevalence of glucose metabolism disorders among HIV-infected adults on long-term ART to ART-naïve adults and HIV-negative controls	153 HIV-negative controls, 151 HAART-naïve and 150 on HAART for 2 years were recruited at an HIV clinic in Mwanza and compared with respect to the prevalence of the outcomes	Glucose metabolism disorders (diabetes, impaired fasting glucose, impaired glucose tolerance)	Additional information such as family history of diabetes, weight change from baseline, were not available.	Patients on long-term HAART had 5-fold greater odds of glucose metabolism disorders than HIV-negative controls.

Continued…

S6 Table Continued…

Author (Year) Country	Study aim(s)	Participants, settings, sampling and methods	Outcomes/findings	Major Limitations	Conclusions
Manuthu (2008) Kenya	To determine the period prevalence of dyslipidemia and dysglycemia in HIV-infected patients	134 patients on HAART and 161 HAART-naïve recruited at a treatment facility in Nairobi. Blood lipids and blood glucose parameters were measured and compared	Dyslipidemia (high triglycerides, high total cholesterol, high LDL, low HDL), Dysglycemia (diabetes, impaired fasting glucose, impaired glucose tolerance)	Adherence to HAART not assessed. Same cut-off value for low HDL used for males and females	HIV-infected patients had a high prevalence of dyslipidemia. HAART use was associated with an atherogenic lipid profile but not associated with low HDL cholesterol and had no significant effect on dysglycaemia.
Mbunkah (2014) Cameroon	To report the prevalence of metabolic syndrome (MS) in Cameroonian HIV-infected on different combinations of HAART and HAART-naïve patients	112 patients on HAART and 61 HAART-naïve patients managed at the Buea and Limbe Regional Hospitals were recruited and parameters measured	Metabolic syndrome (central obesity, elevated blood pressure and glucose, low HDL and high triglyceride)	Few information on risk factors. Low duration of HAART use.	HAART but not HIV disease plays a significant role in the development of MS which may HIV patients to developing cardiovascular diseases and diabetes.
Mohamm-ed (2015) Ethiopia	To assess the magnitude of diabetes mellitus (DM) and associated risk factors in HIV-infected individuals.	Data collected from 260 patient on HAART and 108 HAART-naïve patients attending the Jimma University Specialized Hospital. Blood glucose and lipid profile parameters were assessed	Diabetes Abnormal lipid levels	Difficult to generalize results to rural areas since most of the DM patients were from urban areas.	HAART could have an impact and the cause of diabetes in these patients therefore HIV-infected individuals should be screened for diabetes, both before and after initiation of HAART
Muhamm-ad (2013) Nigeria	To describe the cardiovascular risk profile of HIV/AIDS patients receiving HAART	100 patients on HAART and 100 HAART-naïve patients receiving care at a health facility in northern Nigeria were recruited	Hypertension, diabetes, low HDL, high LDL high triglyceride, high total cholesterol	Only 1 patient was on protease inhibitors which have been noted to affect CVD risk.	HAART treatment was associated with significantly higher prevalences of hypertension, obesity and metabolic syndrome

Continued…

S6 Table Continued….

Author (Year) Country	Study aim(s)	Participants, settings, sampling and methods	Outcomes/ Findings	Major Limitations	Conclusions
Ngala (2013) Ghana	To determine the prevalence of lipid dysregulation and dysglycaemia in HIV infected patients on HAART in the Kumasi metropolis	164 patients on HAART for at least six months and 141 HAART-naive patients were recruited. Blood glucose and lipid levels were measured	Diabetes, Low HDL, High LDL High triglyceride, High total cholesterol	Cut-off values used to define outcomes were not standard.	HAART was associated with lipodystrohy. The risk of developing diabetes mellitus was 5 times higher in the HAART than the HAART naive group.
Nsagha (2015) Cameroon	To investigate the cardiovascular risk profile of HIV/AIDS patients receiving HAART and those not receiving HAART.	160 patients on HAART and 55 HAART-naïve patients were recruited at HIV/AIDS treatment centres in the South West Region of Cameroon	Hypertension Diabetes Abnormal lipid levels	Small number of HAART-naïve patients.	HAART treatment was associated with significantly higher prevalence of hypercholesterolemia, increased LDL and hypertension, hence the risk of cardiovascular diseases.
Ogunda-hunsi (2008) Nigeria	To report the occurrence of hyperlipidaemia in subjects on HAART in a West African Community	55 patients on HAART for a minimum of 3 years, matched for age and sex with 55 HAART-naïve at an HIV clinic	Lipid levels	Small sample size.	The prevalence of hypertriglyceridemia was higher in patients on HAART than in HAART-naïve patients
Ogunmola (2014) Nigeria	To investigates the prevalence of hypertension and obesity and their association with HIV infection and HAART	153 HIV-negative controls, 130 on HAART and 120 HAART-naïve patients were recruited in a rural tertiary health center	Hypertension	Few CVD risk factors assessed. Generalizability of results limited.	HIV or HAART status was not associated with hypertension.
Osegbe (2016) Nigeria	To determine the risk factors and risk assessment for CVD in HIV-positive patients with and without HAART	100 patients on HAART, 100 HAART-naïve and 83 HIV-negative controls attending the Lagos University Teaching Hospital	Elevated blood pressure, Diabetes Abnormal lipid levels	Few CVD risk factors assessed. Generalizability of results limited.	Risk factors and risk assessment for CVD are increased in HIV-positive patients with and HAART

Continued…

S6 Table continued…

Author (Year) Country	Study aim(s)	Participants, settings, sampling and methods	Outcomes/ Findings	Major Limitations	Conclusions
Pefura Yone (2011) Cameroon	To determine the prevalence and characteristics of lipid profile derangements associated with first-line HAART	138 patients on HAART for at least 12 months and 138 HAART-naïve patients were recruited	Abnormal lipid profile	Limited number of CVD risk factors assessed.	First-line HAART is associated with pro-atherogenic adverse lipid profile, necessitating regular monitoring to optimal manage these adverse effects
Tadewos (2012) Ethiopia	To determine the prevalence of dyslipidemia and characteristics of lipid profiles among patients on first-line HAART	113 patients on HAART for a minimum of one year and 113 HAART-naïve patients were recruited in Southern Ethiopia. Serum lipid profiles assessed	Abnormal lipid levels	Small number of male participants. Cardiovascular risk stratifications were not assessed.	Use of first-line HAART containing Efavirenz and Nevirapine were associated with raised total cholesterol, LDL-cholesterol, and triglycerides, an established atherogenic lipid profiles.
Tesfaye (2014) Southern Ethiopia	To estimate the prevalence of metabolic syndrome (MS) among HIV positive patients with and without HAART	188 on HAART and 186 HAART-niave were recruited and parameters measured	Abnormal lipid levels	Limited number of CVD risk factors assessed.	Almost a quarter of HIV patients on HAART developed MS. Patients on HAART had elevated lipid profile. glucose metabolism disturbance than the HAART naïve
