# Supplementary material for: The association between antiretroviral therapy and selected cardiovascular disease risk factors in sub-Saharan Africa: A systematic review and meta-analysis
Source: PLoS One. 2018 Jul 30;13(7):e0201404. doi: 10.1371/journal.pone.0201404 (PMC6066235; doi:10.1371/journal.pone.0201404)
Supplement: S7 Table — (PDF) [file pone.0201404.s007.pdf]

S7 Table. Studies excluded from the review and reasons for exclusion

#	Author (Year)	Title	Reason for exclusion
1.	Abdujalil MM (2015)	Glycaemic and lipid profiles in HIV positive patients on antiretroviral therapy in Sokoto state, Nigeria	- Not comparing HAART+ and HAART- groups<br>- Compared means of outcome variables instead of proportions
2.	Ahomadegbe C (2011)	Cardiovascular risk among people living with HIV at “CNHU-HKM” of Cotonou, Benin	- Conference abstract
3.	Amusa GA (2013)	Lipid Profile of Anti-Retroviral Treatment-Naïve HIV-Infected Patients in Jos, Nigeria	- Not comparing HAART+ and HAART- groups
4.	Berhane T (2015)	Burden of cardiovascular disease risk factors in HIV-infected adults in North-central Nigeria	- Conference abstract
5.	Clark SJ (2015)	Cardio-metabolic disease risk and HIV status in rural South Africa: establishing a baseline	- Not comparing HAART+ and HAART- groups<br>- Data stratified according to sex
6.	Daniyam C (2015)	Assessment of cardiovascular risk factors in people with HIV infection treated with ART in rural South Africa: A cross sectional study	- Not comparing HAART+ and HAART- groups
7.	Isuzuo SA (2009)	Metabolic dysfunctions in non-antiretroviral treated HIV/AIDS patients	- Not comparing HAART+ and HAART- groups<br>- Comparing HIV-infected to HIV negative patients
8.	Kiage JN (2015)	Cardio-metabolic risk factors among HIV patients on antiretroviral therapy	- Not comparing HAART+ and HAART- groups<br>- Studying only HIV/AIDS patients on HAART
9.	Mashinya F (2015)	Assessment of cardiovascular risk factors in people with HIV infection treated with ART in rural South Africa: A cross sectional study	- Not comparing HAART+ and HAART- groups<br>- Studying only HIV/AIDS patients on HAART
10.	Menezes CN (2011)	A longitudinal study of Stavudine-associated toxicities in a large cohort of South African HIV infected subjects	- Not on cardiovascular disease risk factors

Continued…

S7 Table Continued…

#	Author (Year)	Title	Reason for exclusion
11.	Muhammad S (2011)	Effects of haart on cardiovascular risk profile of HIV/AIDS patients in aminu Kano teaching hospital, Kano, Nigeria	- Most recent update of the study included
12.	Muhammad (2013)	Prevalence of dyslipidemia among human immunodeficiency virus infected Nigerians	- Most recent update of the study included
13.	Ngondi JI (2007)	Lipid profile of infected patients treated with highly active antiretroviral therapy in Cameroon	- Compared means of lipid levels instead of proportions with high or low lipid levels
14.	Ngondi JI (2007)	The long-term effect of different combination therapies on glucose metabolism in HIV/Aids subjects in Cameroon	- Compared means of lipid levels instead of proportions with high or low lipid levels
15.	Nsagha DS (2015)	Highly active antiretroviral therapy and dyslipidemia in people living with HIV/AIDS in Fako Division, South West Region of Cameroon	- Most recent update of the study included
16.	Ombeni W (2013)	Lipid Profile in HIV-Infected Patients Using First-Line Antiretroviral Drugs	- Not comparing HAART+ and HAART- groups
17.	Peck RN (2014)	Hypertension, kidney disease, HIV and antiretroviral therapy among Tanzanian adults: A cross-sectional study	- Same results as those reported in the study by Maganga *et al.*, (2015)
18.	Van Rooyen JM (2014)	Cardio-metabolic markers to identify cardiovascular disease risk in HIV-infected black South Africans	- Not comparing HAART+ and HAART- groups<br>- Comparing HIV-infected patients to HIV negative patients
19.	Wembri JP (2008)	A prospective study of 48 weeks in metabolic and anthropometric changes in HIV patients who had Tenofovir containing HAART in a regional outpatient setting	- Conference abstract
20.	Zhou DT (2015)	Dyslipidemia and cardiovascular disease risk profiles of patients attending an HIV treatment clinic in Harare, Zimbabwe	- Compared means of blood pressures and lipid levels instead of proportions with high or low values

HAART+ = HAART group, HAART = HAART-naïve group
