# Supplementary material for: The association between antiretroviral therapy and selected cardiovascular disease risk factors in sub-Saharan Africa: A systematic review and meta-analysis
Source: PLoS One. 2018 Jul 30;13(7):e0201404. doi: 10.1371/journal.pone.0201404 (PMC6066235; doi:10.1371/journal.pone.0201404)
Supplement: S8 Table — (PDF) [file pone.0201404.s008.pdf]

S8 Table. Sub-group analyses and meta-regression for the respective outcomes

Outcome and study Characteristics	Strata	# of Studies	OR (95% CI)	Within-stratum P value (I^2)	Meta-regression relative OR	Between-Stratum P value
**Hypertension**						
Study Quality	Good	5	2.20 (1.16 - 4.16)	0.133 (43.3%)	1.42	0.66
	Fair	3	1.57 (0.38 - 6.40)	<0.001 (91.3%)	1	
Location	West and Central	5	2.20 (1.16 - 4.16)	0.133 (43.3%)	1.42	0.66
	East and Southern	3	1.57 (0.38 - 6.40)	<0.001 (91.3%)	1	
Sample size	≥250	3	1.70 (0.36 - 7.96)	<0.001 (88.9%)	0.86	0.85
	<250	5	1.96 (0.89 - 4.32)	0.007 (71.9%)	1	
Confounders adjusted	Yes	2	1.43 (0.47 - 4.33)	0.142 (53.7%)	0.63	0.61
	No	6	2.15 (0.89 - 5.22)	<0.001 (83.4%)	1	
**Diabetes Mellitus**						
Study Quality	Good	4	1.42 (0.41 - 4.93)	0.163 (41.4%)	0.37	0.38
	Fair	4	4.18 (0.81 - 21.47)	<0.001 (83.6%)	1	
Location	West and Central	5	1.94 (0.74 - 5.08)	0.145 (41.5%)	0.41	0.46
	East and Southern	3	5.26 (0.38 - 73.43)	<0.001 (87.9%)	1	
Sample size	≥250	4	4.99 (0.83 - 30.02)	<0.001 (84.6)	3.49	0.28
	<250	4	1.36 (0.50 - 3.75)	0.271 (23.3%)	1	
Confounders adjusted	Yes	1	0.65 (0.28 - 1.47)	-	0.20	0.26
	No	7	3.29 (1.19 - 9.08)	0.026 (58.0%)	1	
**High Total Cholesterol**						
Study Quality	Good	5	3.90 (2.12 - 7.17)	0.034 (61.5%)	1.02	0.96
	Fair	3	3.83 (1.68 - 8.73)	0.005 (81.3%)	1	
Location	West and Central	6	3.61 (2.03 - 6.42)	0.003 (72.1%)	0.74	0.60
	East and Southern	2	5.01 (3.03 - 8.31)	0.433 (0.0%)	1	
Sample size	≥250	3	3.88 (1.75 - 8.63)	0.005 (80.8%)	0.99	0.99
	<250	5	3.87 (2.07 - 7.24)	0.030 (62.7%)	1	
Confounders adjusted	Yes	1	3.80 (1.61 - 8.95)	-	0.98	0.98
	No	7	3.88 (2.32 - 6.49)	0.002 (71.7%)	1	

Continued…

S8 Table continued…

Outcome and study Characteristics	Strata	# of Studies	OR (95% CI)	Within-stratum P (I^2)	Meta-regression relative OR	Between-stratum P
**High Triglycerides**						
Study Quality	Good	6	1.51 (1.18 - 1.92)	0.507 (0.0%)	1.07	0.73
	Fair	8	1.42 (1.05 - 1.91)	0.189 (30.0%)	1	
Location	West and Central	10	1.34 (1.07 - 1.67)	0.557 (0.0%)	0.806	0.28
	East and Southern	4	1.70 (1.18 - 2.44)	0.149 (43.7%)	1	
Sample size	≥250	6	1.31 (1.06 - 1.62)	0.513 (0.0%)	0.74	0.13
	<250	8	1.76 (1.30 - 2.39)	0.379 (6.6%)	1	
Confounders adjusted	Yes	1	2.50 (1.41 - 4.43)	-	1.84	0.07
	No	12	1.36 (1.14 - 1.63)	0.753 (0.0%)	1	
**Low high density lipoprotein**						
Study Quality	Good	5	0.58 (0.34 - 0.99)	0.020 (76.6%)	1.17	0.77
	Fair	6	0.50 (0.21 - 1.18)	<0.001 (91.9%)	1	
Location	West and Central	7	0.72 (0.38 - 1.35)	<0.001 (86.3%)	2.17	0.14
	East and Southern	4	0.33 (0.20 - 0.54)	0.019 (67.7%)	1	
Sample size	≥250	5	0.65 (0.25 - 1.66)	<0.001 (93.8%)	1.43	0.51
	<250	6	0.45 (0.31 - 0.66)	0.068 (51.3%)	1	
Confounders adjusted	Yes	1	0.44 (0.25 - 0.78)	-	0.80	0.81
	No	10	0.55 (0.32 - 0.95)	<0.001 (88.5%)	1	
**High low density lipoprotein**						
Study Quality	Good	4	2.68 (1.72 - 4.20)	0.158 (42.3%)	1.40	0.58
	Fair	4	2.01 (0.74 - 5.44)	<0.001 (88.5%)	1	
Location	West and Central	5	2.51 (1.55 - 4.07)	0.037 (60.8%)	1.27	0.71
	East and Southern	3	2.03 (0.55 - 7.43)	<0.001 (90.8%)	1	
Sample size	≥250	3	3.91 (2.71 - 5.64)	0.353 (3.9%)	2.33	0.14
	<250	5	1.72 (0.91 - 3.25)	0.002 (75.7%)	1	
Confounders adjusted	Yes	1	2.64 (1.31 - 5.32)	-	1.12	0.90
	No	7	2.35 (1.31 - 4.21)	<0.001 (81.2%)	1	
