# Supplementary figures and images for: The association between antiretroviral therapy and selected cardiovascular disease risk factors in sub-Saharan Africa: A systematic review and meta-analysis
Source: PLoS One. 2018 Jul 30;13(7):e0201404. doi: 10.1371/journal.pone.0201404 (PMC6066235; doi:10.1371/journal.pone.0201404)

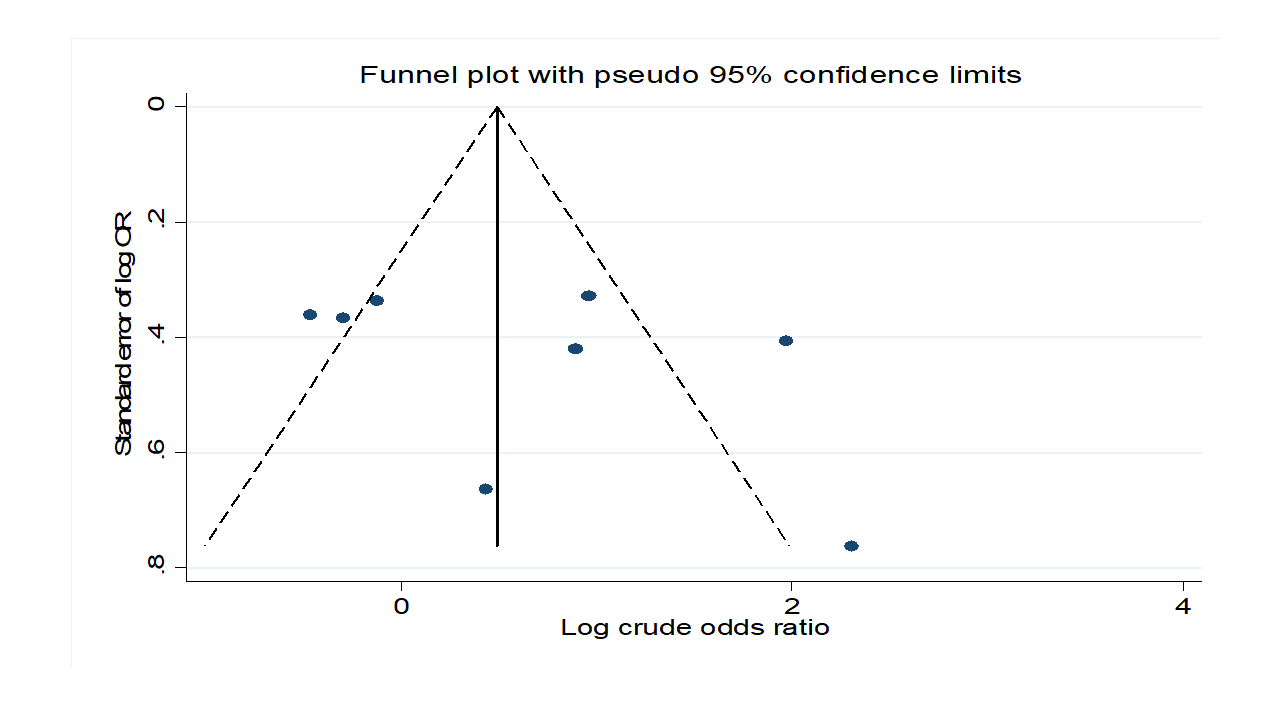

Supplement: S1 Fig — The blue dots represent the studies, the solid vertical line represents the log odds ratio of the pooled estimate obtained from the meta-analysis, the dashed diagonal lines represent the 95% confidence limits around the pooled estimate. (TIF) [file pone.0201404.s009.tif]

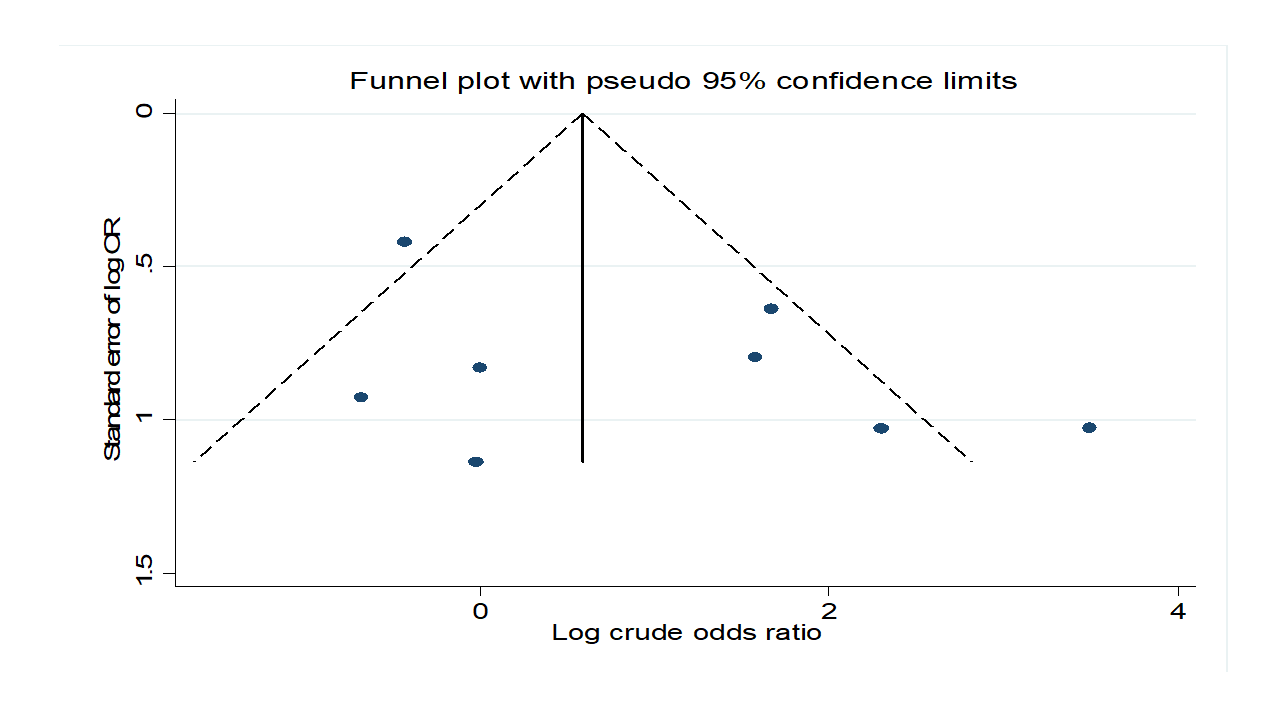

Supplement: S2 Fig — The blue dots represent the studies, the solid vertical line represents the log odds ratio of the pooled estimate obtained from the meta-analysis, the dashed diagonal lines represent the 95% confidence limits around the pooled estimate. (TIF) [file pone.0201404.s010.tif]

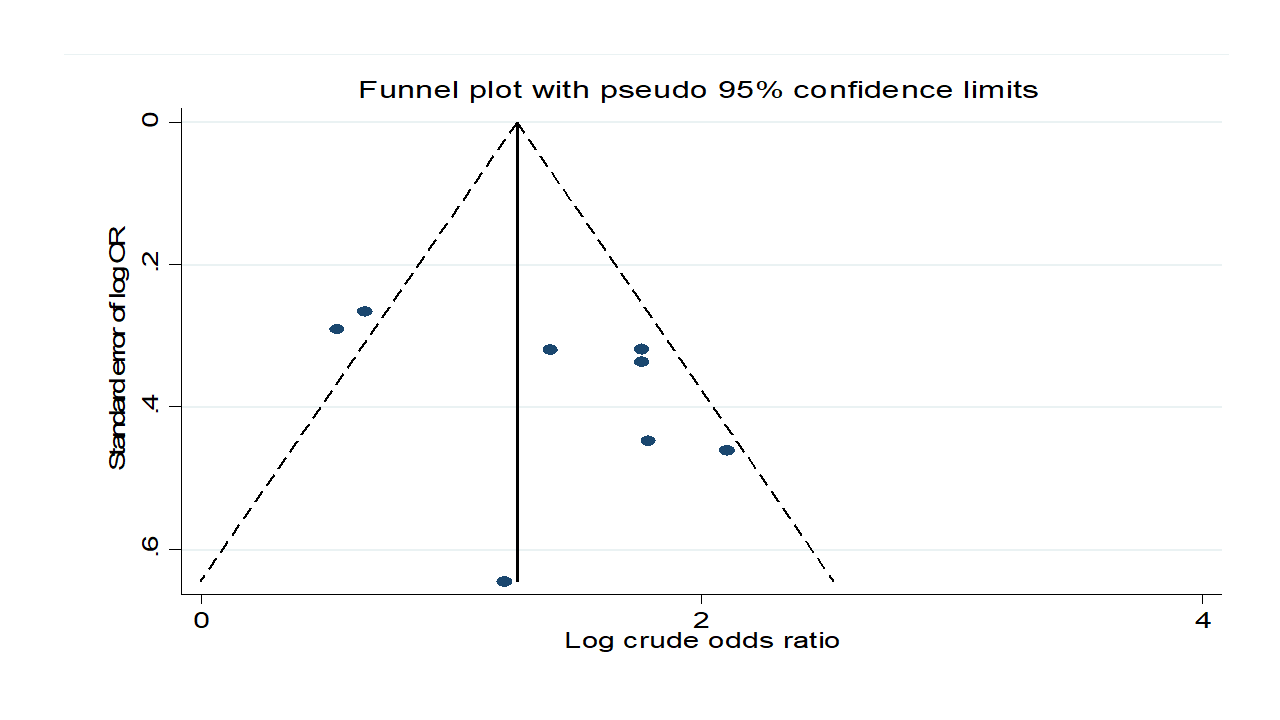

Supplement: S3 Fig — The blue dots represent the studies, the solid vertical line represents the log odds ratio of the pooled estimate obtained from the meta-analysis, the dashed diagonal lines represent the 95% confidence limits around the pooled estimate. (TIF) [file pone.0201404.s011.tif]

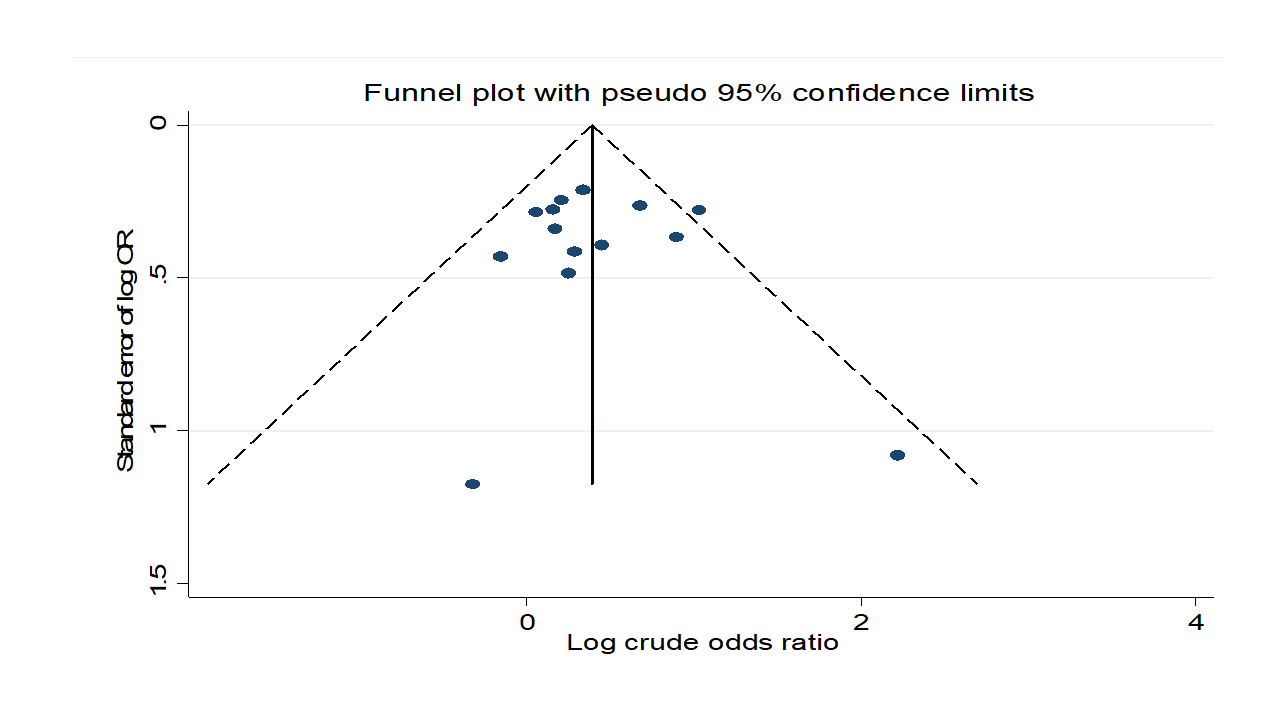

Supplement: S4 Fig — The blue dots represent the studies, the solid vertical line represents the log odds ratio of the pooled estimate obtained from the meta-analysis, the dashed diagonal lines represent the 95% confidence limits around the pooled estimate. (TIF) [file pone.0201404.s012.tif]

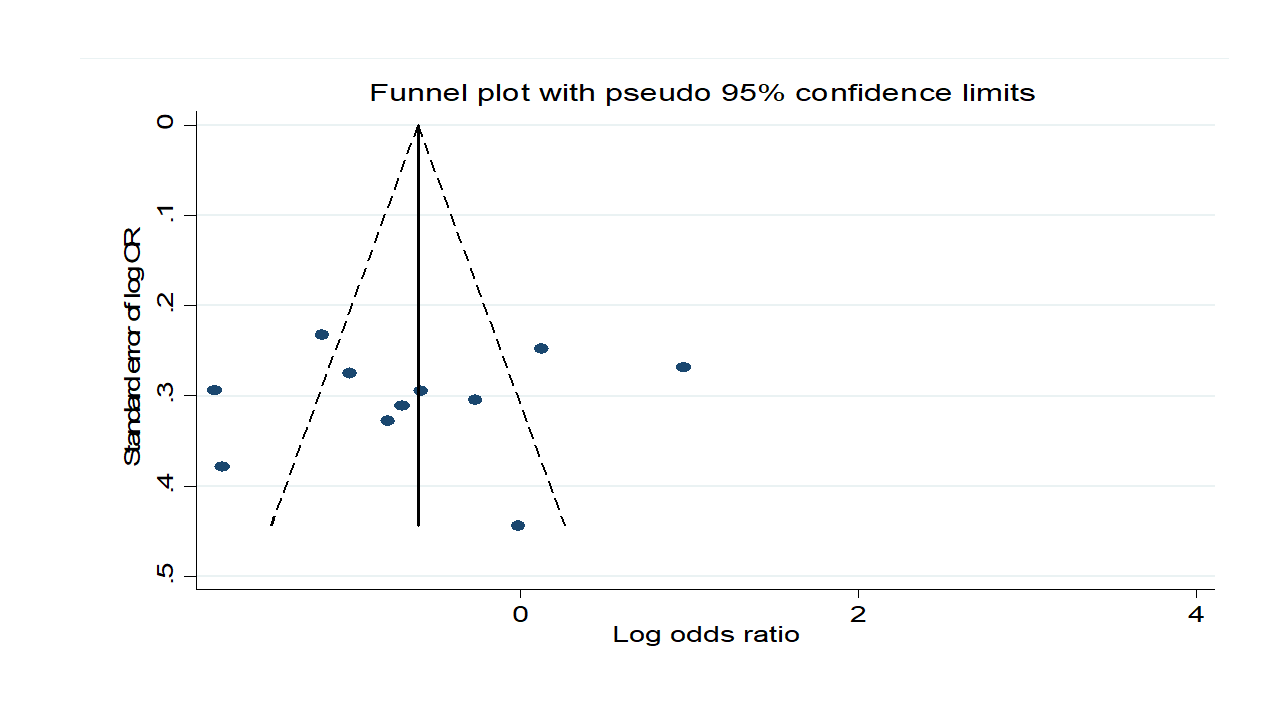

Supplement: S5 Fig — The blue dots represent the studies, the solid vertical line represents the log odds ratio of the pooled estimate obtained from the meta-analysis, the dashed diagonal lines represent the 95% confidence limits around the pooled estimate. (TIF) [file pone.0201404.s013.tif]

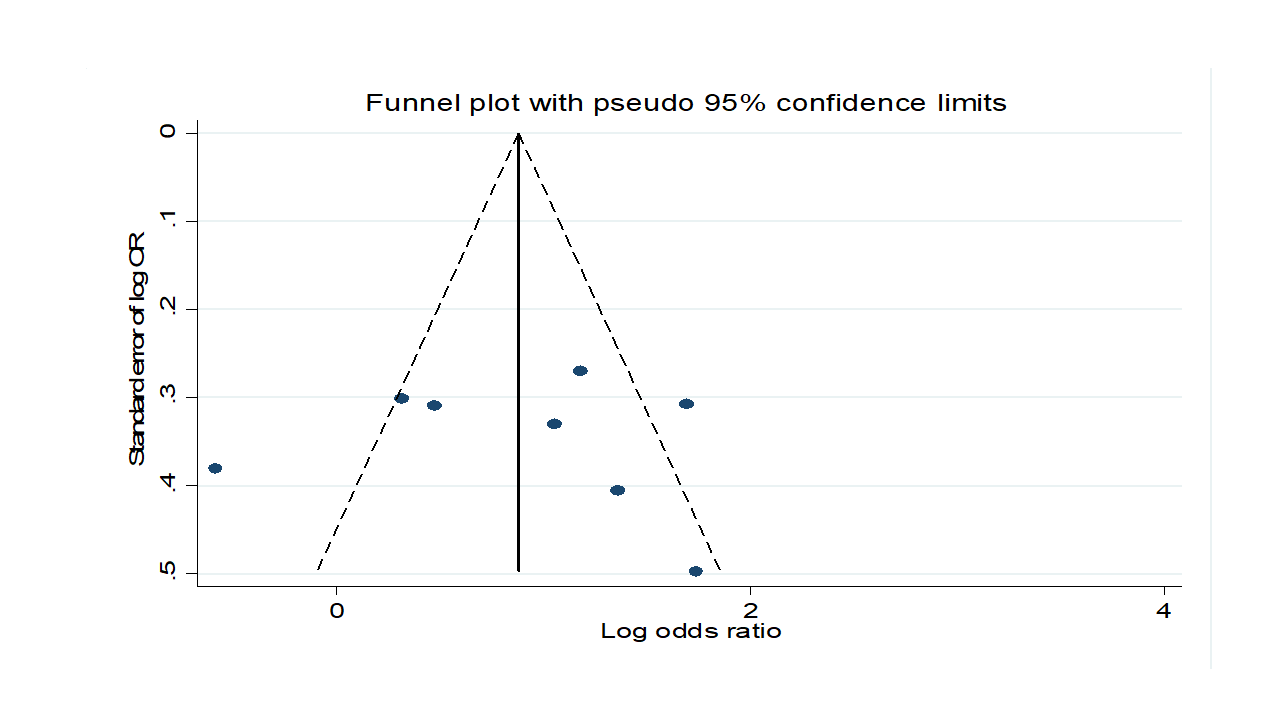

Supplement: S6 Fig — The blue dots represent the studies, the solid vertical line represents the log odds ratio of the pooled estimate obtained using the fixed effects meta-analysis, the dashed diagonal lines represent the 95% confidence limit around the pooled estimate. (TIF) [file pone.0201404.s014.tif]
